# Synthetic data at scale: a development model to efficiently leverage machine learning in agriculture

**DOI:** 10.3389/fpls.2024.1360113

**Published:** 2024-09-16

**Authors:** Jonathan Klein, Rebekah Waller, Sören Pirk, Wojtek Pałubicki, Mark Tester, Dominik L. Michels

**Affiliations:** ^1^ Computational Sciences Group, King Abdullah University of Science and Technology (KAUST), Thuwal, Saudi Arabia; ^2^ Center for Desert Agriculture, King Abdullah University of Science and Technology (KAUST), Thuwal, Saudi Arabia; ^3^ Institute of Computer Science, Christian-Albrechts-University, Kiel, Germany; ^4^ Faculty of Mathematics and Computer Science, Adam Mickiewicz University, Poznań, Poland

**Keywords:** artificial intelligence, data generation and annotation, disease detection, greenhouse farming, machine learning, synthetic data, tomato plants

## Abstract

The rise of artificial intelligence (AI) and in particular modern machine learning (ML) algorithms during the last decade has been met with great interest in the agricultural industry. While undisputedly powerful, their main drawback remains the need for sufficient and diverse training data. The collection of real datasets and their annotation are the main cost drivers of ML developments, and while promising results on synthetically generated training data have been shown, their generation is not without difficulties on their own. In this paper, we present a development model for the iterative, cost-efficient generation of synthetic training data. Its application is demonstrated by developing a low-cost early disease detector for tomato plants (*Solanum lycopersicum*) using synthetic training data. A neural classifier is trained by exclusively using synthetic images, whose generation process is iteratively refined to obtain optimal performance. In contrast to other approaches that rely on a human assessment of similarity between real and synthetic data, we instead introduce a structured, quantitative approach. Our evaluation shows superior generalization results when compared to using non-task-specific real training data and a higher cost efficiency of development compared to traditional synthetic training data. We believe that our approach will help to reduce the cost of synthetic data generation in future applications.

## 1 Introduction

Agriculture globally is more challenged now than ever before, needing to produce more food for a growing human population in the context of accelerating climate change, resource scarcity, and loss of biodiversity. These challenges will require smart, adaptable, and cost-effective technologies, which can maximize yields with minimal resource inputs. To this end, farmers are replacing traditional management practices with highly automated systems. High-tech greenhouses are gaining popularity across the globe, enabling growers to have precise control of crop growing conditions [Ruijs and Benninga (2020); Chow (2021)]; likewise computerized combine harvesters are becoming standard for large-scale open-field farming, removing most of the manual effort required in the open field, significantly increasing yields per labor input [Hassena et al. (2000); Hasan et al. (2019)].

These advanced systems have made farmers increasingly reliant on information and communication technology for management, including wireless environmental monitoring and control systems, remote sensing via unmanned aerial vehicles, and cloud-based farm management software [Mocanu et al. (2015); Messina and Modica (2020)]. The usage of these digital tools has produced large amounts of data that must be efficiently processed, analyzed, and interpreted by the farmer. To address this need, machine learning (ML) has emerged as an essential but still underutilized tool in modern agriculture. Indeed, the practical integration of smart systems powered by ML will be essential to enable agriculture to be maximally resource-use efficient [FAO (2022)].

### 1.1 Main challenges in machine learning

Successfully training a ML model requires three components: (1) the right architecture (i.e., the right type of network for the task and the right way to train it); (2) huge computational resources (depending on the task, whole computer clusters running for several days); and (3) an extensive amount of training data.

Thanks to the continuous research efforts and an active user community, many problems (such as image classification or segmentation) have established architectures that can be readily used [Meta AI (2024)]. Although computational costs can be high for certain projects, service providers exist that provide those with a high flexibility. In practice the biggest remaining factor determining the final performance is the availability of sufficient training data [Mosqueira-Rey et al. (2023)].

Collecting training data from the real world is very costly [Mahmood et al. (2022)]. Not only are many images required, they also need to be diverse and should cover all the variance that the network should learn. For example, if a network is trained on photos taken outside in the summer, it may later perform very poorly on pictures taken in the winter. If an additional use case is added in a later stage, such as also operating during the night time, a large set of new images have to be captured, making these adjustments very costly.

After collecting the training data, it has to be labeled with ground truth information. For some tasks this may be cheap (e.g., assigning the correct class out of a selection of limited choices to each image), but it can still require the work of an expensive expert that can correctly determine the class. For other tasks, such as when pixel-precise segmentation masks should be inferred, the labeling may get very expensive. For the popular *Cityscapes* dataset widely used in autonomous driving, the labeling time required for a single image by an expert ranges from 4 minutes up to 1.5 hours, depending on the density of annotations [Cordts et al. (2016)]. In some cases, data labeling can be outsourced to regions with less expensive labor costs and service providers like *Zuru*, *Cogito Tech*, or *iMerit*, which offer a smooth integration of the process. However, for more complicated labeling tasks such as distinguishing between different kinds of diseases on plants, domain experts may be required for reliable results and then outsourcing becomes infeasible. To some degree, labeling can be sped up through specialized tools (e.g. *LabelBox*). However, such tools can only mitigate the cost; even if costs can be halved, this does not change their order of magnitude.

The most promising candidate for overcoming the excessive cost of obtaining training data is the usage of synthetic training data, which is actually expected to surpass real training data in relevance by 2030 [Gartner (2022)]. Instead of taking photos and labeling them manually, a virtual scene is automatically generated by a computer program and then rendered into a photo-realistic image. The correct label is known from the generation process and requires no additional work while being completely correct. In contrast, manually labeled data almost always includes mistakes caused by human error and generating the labels in the least amount of time using automated strategies sacrifices accuracy [Cabrera et al. (2014)].

Here, the field of computer graphics (CG) comes into play, which researched over the last decades the generation of synthetic renderings including realistic interaction between light and objects [Hughes et al. (2013)]. Using these algorithms, it is possible to generate images that cannot be distinguished from real photographs by humans [Kolivand et al. (2018)]. Using parameterized models, an infinite amount of different images can be created by sampling random parameters, all without additional human work [Kokai et al. (1999)].

Plant modeling has a long history in the CG community. The recursive structure of plants often maps well to recursive algorithm such as *L(indenmayer)-systems* [Prusinkiewicz et al. (2018)] and the elegance of their implementation makes them a very common topic in many introductory computer science lectures [Prusinkiewicz and Lindenmayer (1990)]. Individual plants have been simulated with biological precision to study different phenomena, such as the influence of the canopy to light levels [Chen et al. (2014)]. On a larger scale, the interaction between a large collection of plants and the environment in which they grow has been addressed as well [Marshall-Colon et al. (2017); Makowski et al. (2019)].

Creating powerful parametric models is an expensive task in itself [MacDonald (2018)], but their true power is shown when a scene is adjusted for different scenarios. For example, modeling four different crops at day and night and in summer and winter requires 8 different models but allows for the generation of 16 different combinations. Thus, the cost for increasing the diversity in the training set grows only linearly rather than exponentially.

One way to increase the realism of synthetic renderings is to compute full global light transport using ray tracing [Pharr et al. (2016)]. However, this can immensely increase the computational power required, which directly translates to added costs in hardware and power. In practice, rendering farms can be rented which support parallel rendering on thousands of computers.^^1^^

The distinction between real and synthetic training data is not a sharp one. Real datasets usually contain some form synthetic augmentation (e.g. [Abbas et al. (2021)]), and synthetic renderings are often generated using real images. For example, real photographs can be used as background textures as part of the rendering process [Shorten and Khoshgoftaar (2019)].

### 1.2 Specific contribution

While using real data is hard and costly, using synthetic data also comes with significant challenges. In order to get the most out of synthetic data, the cost delta compared to using real data must be maximized. This type of cost analysis for synthetic data is lacking in the current research. Instead, models are developed without specifying precise targets in performance or cost [Kałużny et al. (2024)], which hinders the practical application and scale-up of this powerful tool.

To address this gap, this study presents a new development model in which synthetic data is generated through an iterative process where each step is guided by a human expert. The task is to estimate in each step what aspects of the renderings have to be improved in order to meet a given target quality without wasting resources on expensive but ineffective improvements. In other words, the goal is to find synthetic datasets that meet the minimal requirements to train successful deep neural network models, as this is the most cost-effective solution.

Using this development model, the potential of synthetic data can be leveraged and significant cost savings reached. After a formal definition of our development model for the general case that includes almost any AI related task, we demonstrate its application and effectiveness addressing a practical use-case of training a neural classifier to distinguish between healthy and diseased tomato plants (*Solanum lycopersicum*) grown in a greenhouse as an example.

## 2 Related work

ML has demonstrated a wide range of applications in the agricultural domain, including the management of crops, livestock, soil, and water. A comprehensive literature review of ML applications in agriculture shows that research has primarily focused on crop management [Benos et al. (2021)]. Within this domain, ML techniques have been applied extensively to yield prediction [van Klompenburg et al. (2020)], crop recognition [Horng et al. (2020)] and harvesting [Wouter Bac et al. (2017)], as well as weed detection [Wang et al. (2019)].

A large body of ML research in crop management focuses on disease detection in plants [Benos et al. (2021)]. This focus on disease detection is well-justified, as pests and diseases are a major challenge for agriculture and food security globally, causing an up to 40% loss in yields each year [Savary et al. (2019)]. Early disease detection in agricultural crops enables earlier interventions that can prevent spread, saving substantial amounts of time and resources. Mitigation measures are generally more effective if applied at the early stages of disease, which also results in less pesticide used for management of the pathogen. Commercial agriculture currently relies on skilled human scouts for disease detection. Ideally scouts do daily walk-throughs, but due to costs and limited personnel, walk-throughs are typically much less frequent in practice. Manual detection methods are neither quick nor failsafe – detecting symptoms in crops requires careful attention, especially in the early stages, and costly errors are sometimes made.

Considering these challenges in manual disease detection, much attention in the past two decades has been directed to automated methods of detection, which utilize optical sensors to survey the crop and support in detection and diagnosis of plant diseases [Mahlein (2016); Chin et al. (2023)]. Tools such as RGB, multi- and hyper-spectral, thermographic, chlorophyll fluorescence, and 3D imaging sensors are able to measure changes in plant physiology as the plant experiences biotic stress from disease. Common symptoms of disease in plants include leaf malformation, discoloration, and wilt. These can be detected via changes in plant or leaf temperature, reflectance, and fluorescence.

Despite advances in sensing technologies in recent decades, there are numerous challenges which limit the scope of automated disease detection applications. A main challenge is the selection of the appropriate image features (i.e. texture, color, and/or shape) which have to account for the complexity of various symptoms as well as the capturing modality that can be performed throughout the growing area [Barbedo et al. (2016)]. Another challenge is the development of accurate and efficient learning algorithms. Accurate classification of diseased and healthy plants in real conditions with varying light levels, shading, and complex surroundings can be extremely difficult [Reddy et al. (2022)]. In addition, large image datasets in a diversity of conditions are needed to train the algorithm. *PlantVillage* is the largest and most widely studied repository of real images of diseased and healthy leaves [Hughes and Salathe (2015)], but its usefulness is limited by the fact that all of the images are segmented leaves with a homogeneous dark background.

Gathering real images of diseased plants at different stages of infection, but particularly at early stages of infection, is an often a challenge because of lack of available data. This challenge was demonstrated in in [Wspanialy and Moussa (2016)], where a system for early detection of powdery mildew disease in greenhouse tomato in a natural setting using a camera setup with varying light settings is developed using Hough forests as the detection algorithm. According to the authors, the study was limited by the size of the dataset (60 images in total) that could be used for training and testing the classifier model.

Synthetic data is a promising solution for the lack of sufficient and high-quality, real training data for ML tools, and in recent years has been explored for agricultural applications [Barth et al. (2018); Cieslak et al. (2024)]. Augmentation (i.e. applying various geometry and color transformation) of real images can be understood as a ‘proto-synthetic’ approach. For example, [Pearlstein et al. (2017)] use an image dataset of grass with and without weed incidence to train a neural network on weed detection. The authors apply a custom software to augment real images of a lawn, which were captured with a smartphone mounted on a robotic vehicle.

*Generative adversarial networks* (GANs) can be used as an even stronger form of augmentation. [Chen and Wu (2023)] developed an 3-stage deep-learning pipeline for detection of grape leaf disease by applying a deep-convolutional GAN to generate partial images of lesions on leaves for training. In total the GAN generated a dataset of 3390 augmented lesions based on 850 real and manually augmented lesions, that could then be identified by a residual neural network, achieving 88% accuracy on a random dataset of 100 real labeled images from the Internet. [Arsenovic et al. (2019)] use a GAN to supplement traditional augmentation techniques to create an image dataset called *PlantDisease* for leaf diseases in more real-life conditions, as an alternative to the *PlantVillage* dataset. [Abbas et al. (2021)] utilize a *conditional* GAN (C-GAN) for image augmentation of diseased and healthy singular tomato leaves (also called “leaflets”) from the *PlantVillage* dataset. Their model achieved high accuracy (*>* 97% mean average precision), but improving it further is made difficult by the limited amount of available input data for the GAN.

Today’s generative AI has however severe limitations: While neural style transfer can be used to give renderings a more photorealistic look, they can only change colors and shading of objects, not their shape. Image generation network on the other hand can generate novel scene perspectives, but only if they are part of their training data. For these reasons, [Kałużny et al. (2024)] use a two-step approach: A procedural model creates the scenes including objects, their shape, and camera perspective, after which a rendering of this scene is improved through style transfer. The current limitations of generative AI are thus overcome through procedural modeling.

Synthetic data often suffers from the so called *domain gap*, as they are systematically different from real data (e.g. renderings looking artificial rather than photo-realistic). Overcoming this domain gap is an important step to enhance training results and has been extensively studied in the past [Sankaranarayanan et al. (2018); Tremblay et al. (2018)].

[Wouter Bac et al. (2017); Barth et al. (2018)] demonstrated for the first time the use of fully synthetic training data in a computer vision task in the agricultural domain when they created a synthetic image dataset of sweet peppers in a greenhouse. The authors used a few real images captured by a harvesting robot as a template to build a model based on *PlantFactory* that generates randomized instances of the plants, fruits, and backgrounds. These scenes are then rendered using *Blender*, requiring about 10 minutes of rendering time per scene on state-of-the-art hardware. With these images, it was for the first time possible to train a neural network for the segmentation of anatomical plant components without relying on excessive real data. However, considering the high amount of computational time necessary to generate the synthetic dataset, the authors point out the need for a more optimized process.

Synthetic training data does not need to be limited to static images. Physic simulations can generate video clips or time resolved 3D position data of moving plants, e.g. a harvest robot pulling off a fruit. This data can then be used for training purposes, avoiding the need to repeat countless real measurements [Deng et al. (2024)].

## 3 The synthetic data pipeline

**Figure 1:**
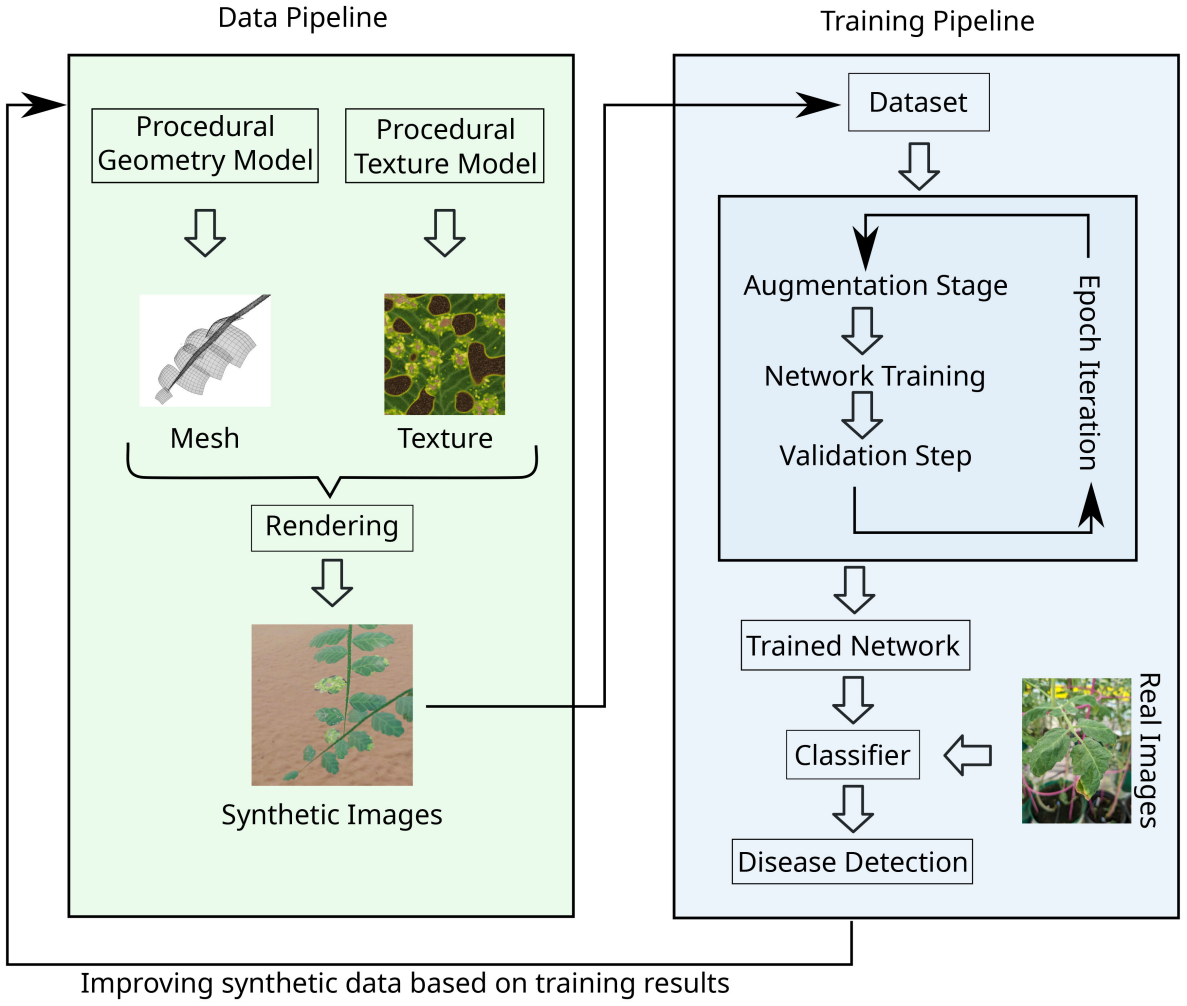
Illustration of our synthetic data pipeline. Left: Geometry and textures are generated, and used to render synthetic scenes. For each of them a procedural model is developed that can create an arbitrary amount of instances sharing the same general appearance. The rendering is then performed with any 3D rendering software. Right: A dataset of synthetic renderings is used to train a network. The standard loop of augmentation, weight training and validation is performed, resulting in a trained neural network that classifies images. Based on the evaluation of this classifier, the data generation is improved and the dataset regenerated for the next iteration.

Our method is described in **Figure 1**: We first define procedural models that generate the geometry of plants and accompanying textures required for rendering. We then use procedural models to generate scenes of tomato plants in a greenhouse setting and render photorealistic images. Each image is associated with a label, that defines whether or not a plant is diseased. A set of rendered images along with their labels is then used as a dataset for training the classification neural network. During training, data augmentation via various image-based operators (e.g. brightness, contrast, etc.) is applied to the input images to increase the variance of images in the training dataset. After the classification network has been trained, we validate its performance based on a dataset of real images. The resulting performance is analyzed qualitatively and quantitatively by a human expert to determine how to improve the procedural models for the next training cycle. Overall, the generation process of synthetic datasets is complex and goes along with the repeated training of the network.

### 3.1 Network training

**Figure 2:**
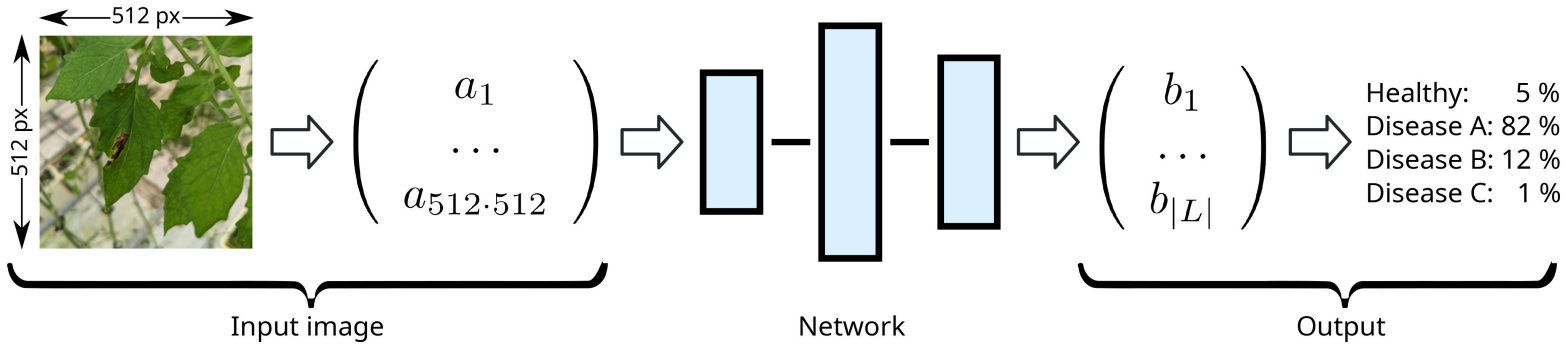
Illustration of ML driven classification. The input image is encoded in an input vector (which can have a very large number of components and represents the pixel color values) that is then can be processed by the different neural network layers, where the result of each layer is the input for the next one. The last output vector is then decoded (e.g. from a negative log-likelihood encoding), resulting in a probability per class.

Since networks are mathematical objects, the input image has to be encoded into a vector of real numbers first. This high-dimensional vector is then processed by the network and transformed into a low-dimensional output vector which can be decoded into the classification. We call the vector space of encoded input images *I*, and an individual input image i∈I . R⊂I is the subspace of real input images, while S⊂I is the subset of synthetically generated input images. *L* is the space of all possible labels, e.g., *L* = {healthy, infected} in case of a binary classifier distinguishing between healthy and infected plants. Such a classifier is illustrated in **Figure 2**.

In a dataset D:=D(J) = {(i,G(i))|i∈J)}⊂D:=I×L , each image i∈J⊂I is assigned a ground truth label G(i) via G:I→L . While *G* maps any image (whether real or synthetic) to its correct label, *D* consists only of a limited amount of images and their labels. The number |J| of images in *J* is typically in the range between a few thousands and a multiple of ten thousand. A network $N_{w}:I\to L,N\left(i\right)\;\mapsto p$ , which is parameterized by its weights *w*, similarly maps images to predictions p . If p=G(i) , the prediction is correct.

The training is influenced by several parameters as well, called the hyper parameters h∈H . Note, that *h* denotes a vector containing all hyper parameters and accordingly *H* is the set of all possible hyper parameter combinations. The hyper parameters include the learning rate and batch size of the stochastic gradient descent optimizer, and also data augmentation parameters (see below) which have a significant impact on the learning success.

The training function $T:H\times D\to \;\left(I\to L\right),T\left(h,D\right)\;\mapsto N_{w}$ then maps a combination of dataset and hyper parameters to a trained network $N_{w}\in \;\left(I\to L\right)$ with weights w∈ ℝ . The mapping ‖·,·‖:L×L→ℝ is a measure of similarity between two labels. We can express *T* as:


(1) $$T\left(h,D\right)=N_{w},\text{ where }w=\underset{w^{\prime }\in \mathbb{R}}{\arg\;\min}\sum_{i\in D}\left\|N_{w^{\prime }}\left(i\right),G\left(i\right)\right\|.$$

Overfitting is often the result of a lack of diversity in the training data. This can either mean too few input images, or images that are too similar to each other (e.g. showing different objects always from the exact same angle). A common strategy to mitigate overfitting is data augmentation. During data augmentation, random alterations are performed on the image, such as geometric transformations (e.g., mirroring, rotating, zooming), color adjustments (brightness, contrast, hue), or adding noise. More advanced augmentation methods use neural networks to transform an input image into an entirely new, but similar one [Abbas et al. (2021)]. A thorough overview of augmentation strategies is found in the literature [Shorten and Khoshgoftaar (2019)]. Since augmentation increases the diversity, it can actually reduce the network performance on the training dataset. This is however acceptable, since at the same time the performance on new images is increased. In summary, two key factors are important for a successful training: A training dataset with a large variety and the correct hyperparameters for the training. The framework presented in this paper optimizes both of them.

### 3.2 Data generation

**Figure 3:**
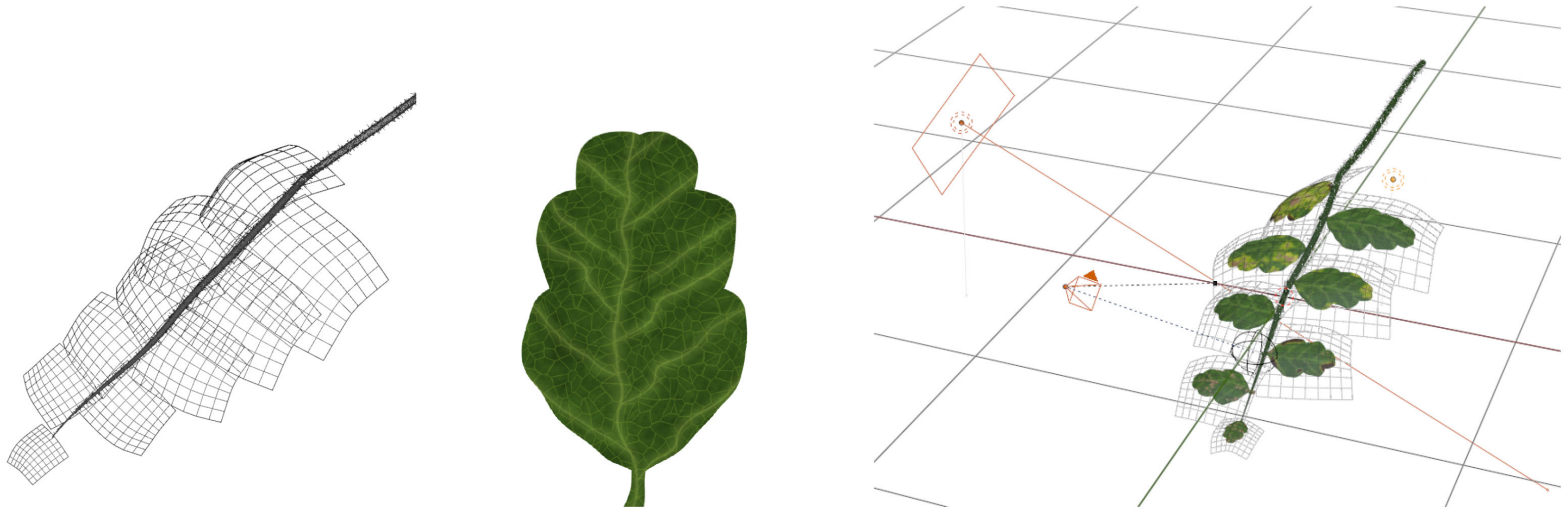
Illustration of the main components of a synthetic scene. Left: Object geometry, represented as a triangle mesh. Middle: Materials, represented as a set of textures (ambient, normal, reflectivity, etc.) Right: Scene composition, where geometry and materials are combined and lighting and camera information are added.

Synthetic dataset are created by means of CG methods. CG is an extensive research field with a rich history [Hughes et al. (2013)] dealing with the modeling and rendering of virtual scenes. The three main components that need to be modeled are geometry, materials, and scene composition (object positions, lighting, camera). Examples are shown in **Figure 3**.

All these components can either be created by an artist by hand or automatically generated through a procedural model. Instead of defining properties (such as the outline of a leaf or the branching structure of a plant) by hand, procedural modeling defines rules that depend on various parameters, and can be instantiated to create geometry. An example of such a procedural model in the agricultural context are L-systems for plant geometry and node-based texture synthesis of materials [Pai (2019)]. By varying the input parameter vector, and endless amount of images can be created. However, this does not mean that the diversity is sufficiently high. The instantiation of procedural parameters essentially resembles an interpolation. If a larger portion of the total image space *I* should be covered, additional parameters must be added.

## 4 Using synthetic data at scale

The main bottleneck of the procedure described in the previous section is the generation of a suitable training dataset *S* and the finding of the correct hyperparameters *h* at minimal development cost. The cost factor deserves special attention here since the main argument for the use of synthetic training data is their cost effectiveness. In order to use synthetic data at scale, i.e., being able to apply the previously described procedure for a large number of automation tasks in agriculture, we introduce a development model that addresses this problem by taking a holistic approach. Like the network training itself, finding *S* and *h* is formulated as an iterative optimization scheme where in each step *S* and *h* are gradually improved.

The first step is to determine a suitable target quality $q_{\text{min}}$ . We measure the performance q=[N(R),G(R)] of a network *N* on a real dataset R⊂I using a measure $\left[\cdot ,\cdot \right]\;:L^{\left|R\right|}\times L^{\left|R\right|}\to \mathbb{R}$ which is, e.g., defined as the *F* score or the *P*_4_ metric in case of binary classification [Manning et al. (2009)]. Without loss of generality, we assume that larger values of *q* are better. Note, that the network is trained on annotated synthetic data D=D(S) but evaluated on real data *R*. Our goal is then to find a pair (h,S) ⊂H×I such that


$$\left[T\left(h,D\left(S\right)\right)\left(R\right),G\left(R\right)\right]=:q\geq q_{\min}.$$

Without consideration of any cost, *h* could be found by a brute-force parameter sweep, while for *S* renderings with the highest degree of photorealism and variability could be used. But if we assume that the quality is proportional to the invested cost, the goal becomes finding the worst pair (h,S) which still satisfies $q\geq q_{\min}$ . We address this iteratively. An iteration step


$${ℐ}_{k}:H\times D\to H\times D,\left(h_{k},S_{k}\right)\mapsto \left(h_{k+1},S_{k+1}\right)$$

refines the hyperparameters and the dataset (though it is not required that both change in each iteration). Each iteration ${ℐ}_{k}$ is associated with a certain cost measured by the cost function $C\;\left({ℐ}_{k}\right)$ . The overall optimization problem is then to find the sequence $\left({ℐ}_{1},\ldots ,{ℐ}_{n}\right)$ of iterations with minimal cost that yields the desired quality:


(2) $$\underset{\left({ℐ}_{1},\ldots ,{ℐ}_{n}\right)}{\arg\;\min}\sum_{k=1}^{n}C\left({ℐ}_{k}\right),\text{ subject to }q\geq q_{\text{min}}.$$

Here we see why a reasonable choice of $q_{\text{min}}$ is important: According to the Pareto principle, if $q_{\text{min}}$ is too large, this results in an excessive amount of iterations with exponentially growing costs. Knowing what quality is acceptable is crucial to minimizing cost. Solving Equation 2 cannot be performed automatically through naive numerical optimization. Rather, every iteration step *k* requires the guidance of a human expert. The solution is typically obtained by maximize the quality gradually at every step.

Taking a closer look at $C_{k}:=C\left({ℐ}_{k}\right)$ , shows that it consist of several components:


$$C_{k}=C_{k}^{\text{E}}+C_{k}^{\text{M}}+C_{k}^{\text{R}}+C_{k}^{\text{T}},$$

where $C_{k}^{\text{E}}$ is the cost of evaluating the previous iteration required for deciding on the next changes, $C_{k}^{\text{M}}$ is the modeling cost to improve the generator for the synthetic images (performed by an artist), $C_{k}^{\text{R}}$ is the required rendering costs for the new dataset (often outsourced to a rendering farm and paid per core minute) and $C_{k}^{\text{T}}$ is the cost of training a new network with the improved hyperparameters and dataset. The cost for changing the hyperparameters is entirely contained in $C_{k}^{\text{E}}$ , since they are a simple vector that requires no modeling time. For a brute force search of the best hyperparameters the total cost is dominated by $C_{k}^{\text{T}}$ since the dataset remains the same $\left(C_{k}^{\text{M}}=C_{k}^{\text{R}}=\;0\right)$ and $C_{k}^{\text{E}}$ is minimal (as it only consist of a sampling strategy for *h*). Often the most expensive step is to improve the dataset since $C_{k}^{\text{M}}$ and $C_{k}^{\text{R}}$ are typically large. It can be beneficial to split such an iteration into multiple sub-iterations, which introduces additional $C_{k}^{\text{T}}$ , but gives an overall better understanding of the required changes.

## 5 Case study: early disease detection for tomato plants

The previously described development model is now applied in order to develop a neural classifier for early disease detection of tomato plants (*Solanum lycopersicum*). This use-case is not only suitable to demonstrate our development model but also addresses an important practical problem. Especially in monocultures found in greenhouses, diseases can spread rapidly and can quickly become uncontrollable [Savary et al. (2019)]. Detecting them as early as possible greatly decreases the chance of such a catastrophic crop failure but requires constant and expensive monitoring. Any step toward automatizing this process is therefore a great benefit.

**Figure 4:**
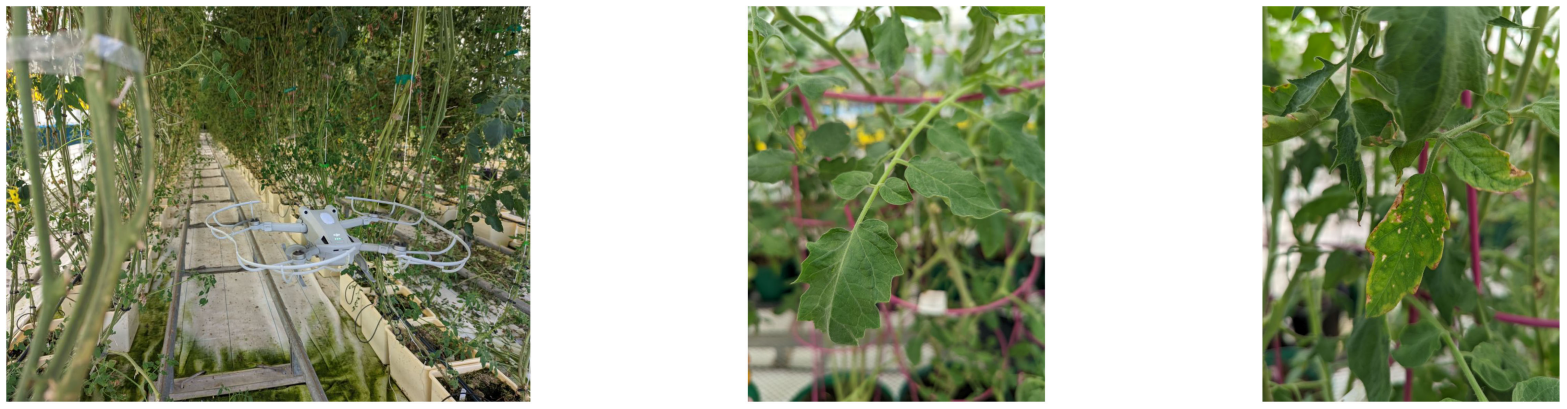
Illustration of the image collection process in the greenhouse complex hosting tomato plants (*Solanum lycopersicum*). Left: An autonomously flying UAV patrols through each line, taking photos. Middle: Example of a healthy leaf. Right: Example of an infected leaf.

We use a UAV patrolling through rows of the greenhouse complex in order to capture images of the tomato plants as illustrated in **Figure 4**. Since the tomato plants may grow to lengths of 40 m over the course of a season, we prefer to use UAVs instead of self-driving vehicles patrolling through rows. This also comes with low hardware costs as the price of our *DJI Mini 3 Pro* is below USD 1000. This UAV is also sufficiently small in size to fly through the rows of the greenhouse. For larger greenhouse complexes, multiple UAVs can be used, e.g., a single drone per row. Note, that this is an illustration to motivate our research topic. The case study is focused on visual disease detection and not on drone control, and the pictures shown throughout this paper were taken manually. An overview of drone control techniques is given in [Merkert and Bushell (2020)].

In this section, we focus on the binary classifier which groups pictures of the leaves into two classes containing healthy and infected leaves (|L| = 2) . Potential infections can then be reported to a human overseer who can confirm or reject them. Reducing the need for manually checking the entire greenhouse complex to checking only a few candidates greatly reduces cost even if the detection rate is not perfect. Overall we aim for an accuracy of $q_{\min}\approx \;90\%$ .

**Figure 5:**
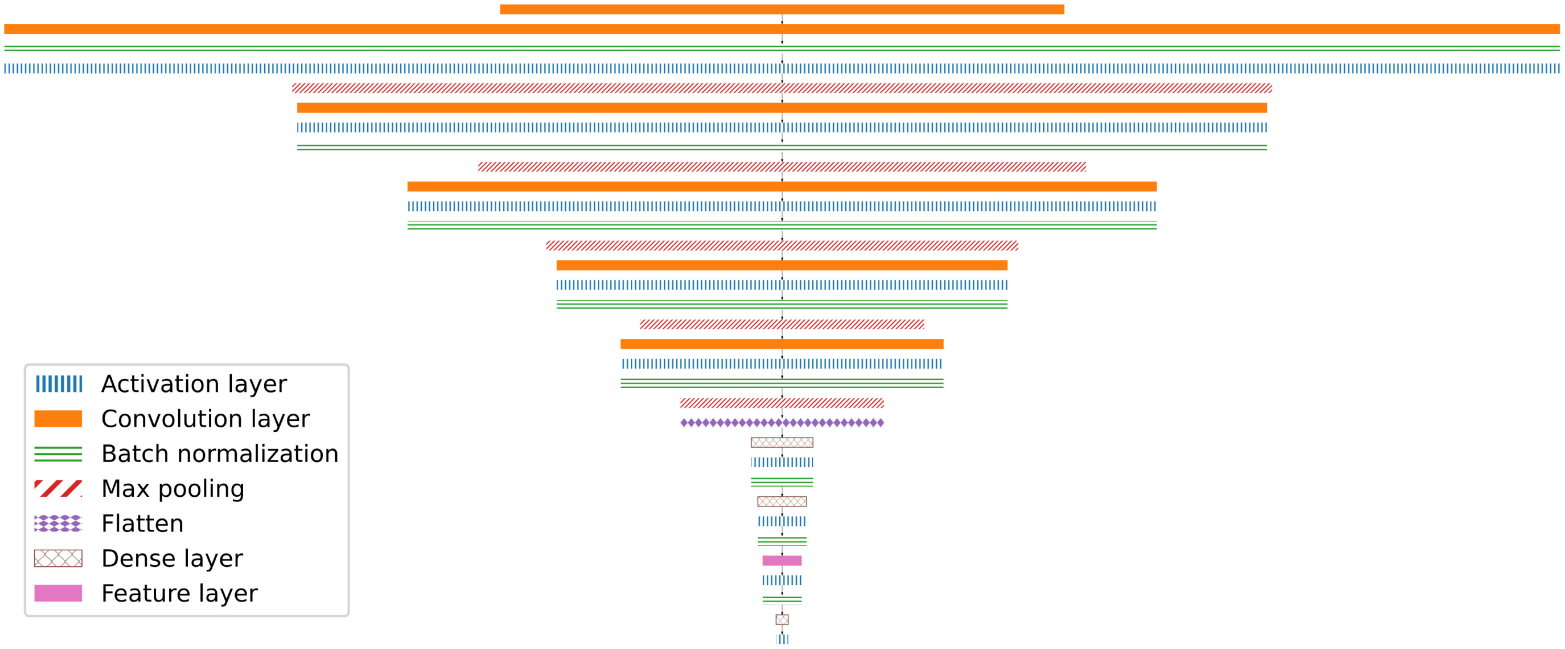
Simplified illustration of the layered architecture of our classification network. Each layers width corresponds to the cubic root of its dimensionality. The input image (top row) is expanded into multiple parallel filters and throughout the network their size consecutively shrinks until a single value denoting the classification remains. The total number of weight in this network is |*w*| = 2960514. The exact network definition is given in **Appendix 1.3**.

We implement our neural classifier in *Keras* using a state-of-the-art image classification architecture [Géron (2019)]. On overview of the network architecture is shown in **Figure 5**; the exact definition is given in the **Appendix 1.3**. To measure the training loss ‖·,·‖ we use the *categorical cross-entropy* loss function as implemented in *Keras*. As a performance measure [·,·] , we divide the number of falsely labeled images by the total number of images. We start with an initial choice of *h*_1_ and a simple initial dataset $D_{1}:=D\left(S_{1}\right)$ and refine it over the course of a total of n= 6 iterations generating $D_{2}:=D\left(S_{2}\right),\ldots ,D_{6}:=D\left(S_{6}\right)$ and hyperparameter $h_{2},\ldots ,h_{6}$ to reach our target accuracy. After each iteration, an extensive evaluation is required to make an informed decision about the next changes in *h* and *D* (which is the reason why this evaluation is included as the cost $C_{k}^{\text{E}}$ ). We monitor the achieved performance on the synthetic training and validation datasets as well as on a real dataset *R*. Moreover, we take a closer look at the performance on individual images which helps us to understand what additional features have to be modeled in the synthetic images.

### 5.1 Iteration k=1

**Figure 6:**
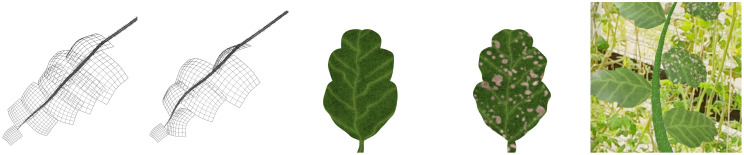
Illustration of geometry and textures of the first iteration of synthetic data. Left: The geometry of the branches is procedurally generated and two examples are shown. Middle: Textures for healthy and infected leaves are generated. The infected textures are generated from the healthy ones by adding typical patterns of dead leaf cells. Right: A final rendering of a textured branch in the scene.

The initial dataset is shown in **Figure 6**. To generate synthetic plant geometry, we have implemented a node-based procedural modeling system as commonly used L-systems for tomato plants [Chen et al. (2014)] do not aim for the level of realism required for our task. The model has a large number of parameters including the number, size, and orientation of leaves, as well as bending and length of the branch. The leaf textures are generated using *Adobe Substance 3D Designer*, see also **Appendix 1.1**. We generate the typical set of physically-based rendering (PBR) textures which include layers such as a diffuse albedo map, a normal map, an ambient occlusion map, and a height map [Hughes et al. (2013)]. With these layers, we do not only model the color of the leaves but also the physical interaction of light with the leaf material, which greatly enhances realism. The scene consists of a single branch with leaves and a random high dynamic range (HDR) panorama photo captured in the greenhouse as background, see also **Appendix 1.2**. This panorama also illuminates the scene, meaning that it is illuminated by the same lighting conditions as the plants in the greenhouse. Although the generated renderings look plausible and detailed, they do not look completely photorealistic. A human may initially be fooled to think they are real images, but in comparison with actual photographs the differences become visible.

**Figure 7:**
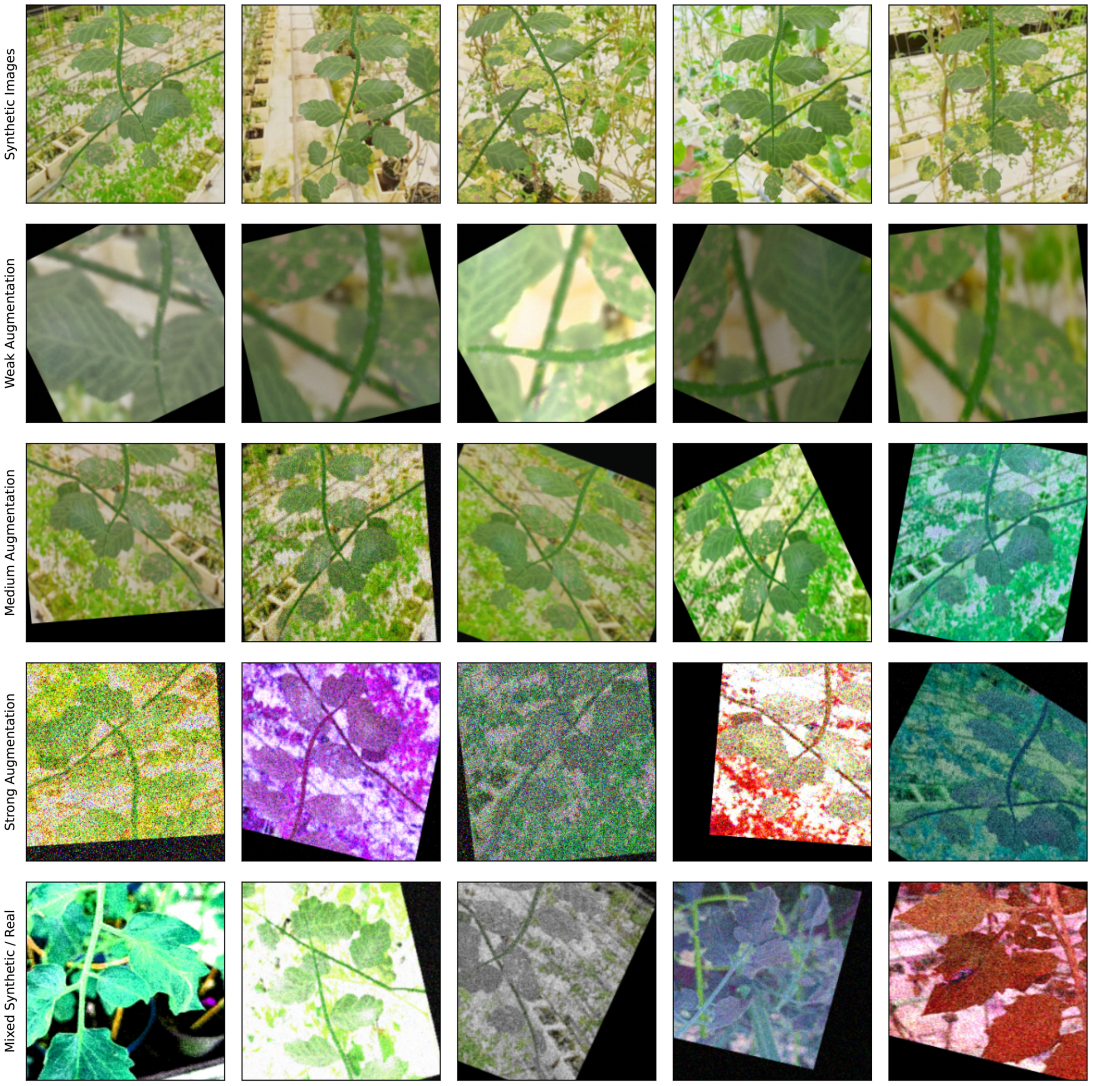
Comparison of augmentation modes. First row: Five different synthetic renderings. Second to fourth row: The first image of the first row augmented 5 times for each of the 3 augmentation modes (weak, medium, and strong). Fifth row: A random selection of real and synthetic images augmented with strong augmentation. Through the augmentation process, it becomes difficult to distinguish between real and synthetic images, thus the domain gap between both sets is reduced.

For the hyperparameter *h*_1_ we chose values typical for a binary classification task: The input resolution is 256 × 256, the batch size is 16 and the learning rate is 10^−4^. For the augmentation, we chose a simple combination of zooming, brightness adjustment, flipping, and rotation. Examples of augmented images are shown in **Figure 7**.

**Figure 8:**
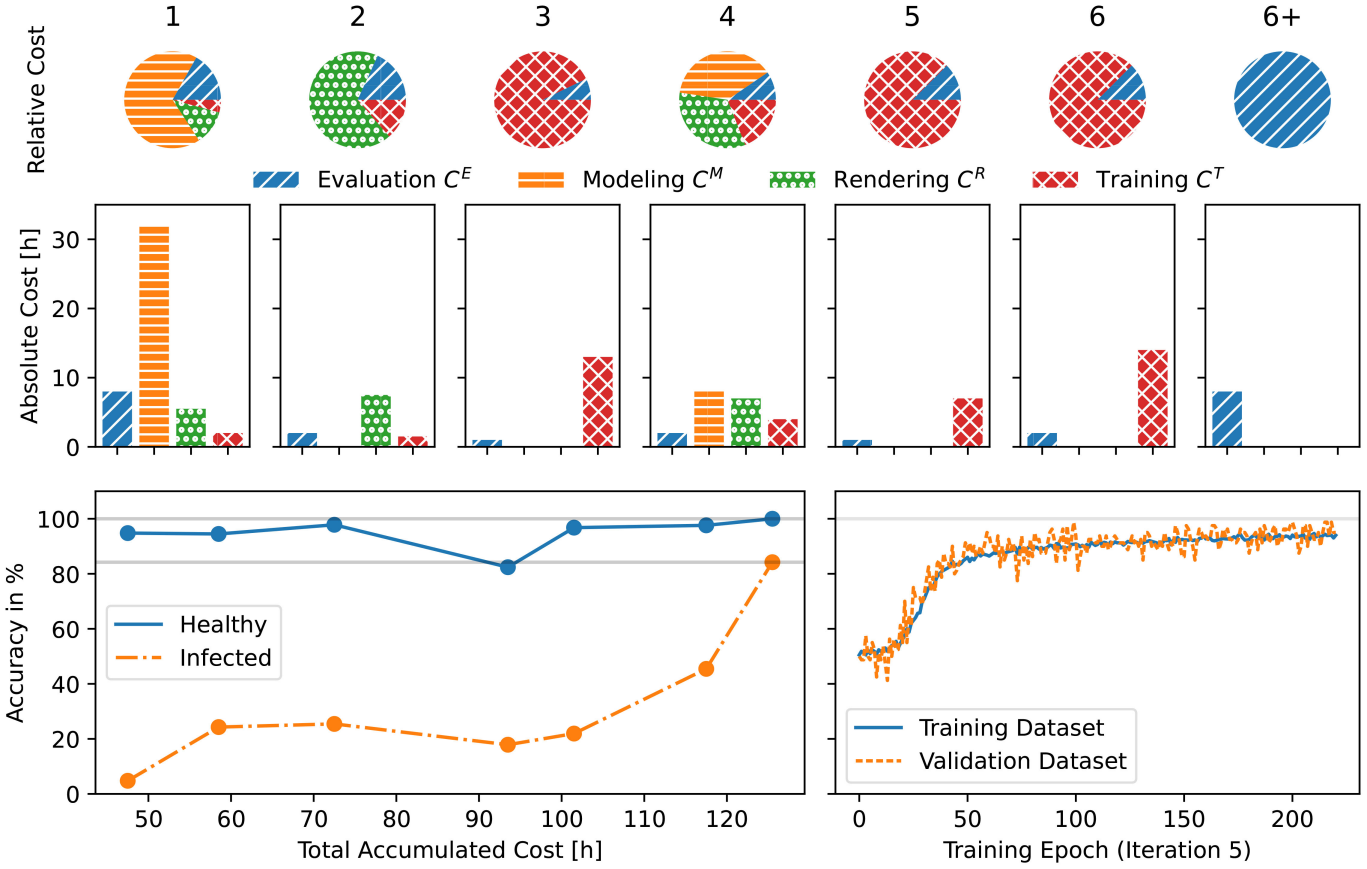
Development cost and performance of each iteration. Top row: Relative cost of the different cost types for each iteration. Middle row: Absolute values of the cost in hours. $C_{1}^{E}$ includes the initial development of the classifier framework. $C_{6+1}^{E}$ include the statistical analysis described in Section 5.7. Bottom left: The accuracy q= [T(h,D(S))(R),G(R)] of the classifier plotted over its development time. The dots mark the individual iterations. Bottom right: The training process of iteration 5 shows, that the accuracy on the validation dataset closely follows the accuracy on the training dataset indicating that the network generalizes well. The lines show the average of the healthy and infected classes. Note, that the lowest accuracy for a binary classification is 50% which corresponds to random guessing of the class.

The initial dataset consists of $\left|S_{1}\right|=\;3400$ images, where half of them show healthy and half of them show infected leaves. Around 10% of the images are used for the validation dataset. Monitoring the performance on both synthetic datasets shows that the network does not overfit as shown in **Figure 8**, bottom right. This means, that the dataset is sufficiently large and by adding more images we likely would not see an increase in quality. This information helps to cap the cost $C_{1}^{\text{R}}$ .

We also evaluate the performance on real data as shown in **Figure 8**. We find that almost all images are classified as healthy regardless of their actual class which means that we learn almost nothing about the true class.

### 5.2 Iteration k=2

After reviewing the results we conclude that the size of the dataset, the network architecture, and training hyperparameters are fine (since the accuracy on the validation dataset is high), but that the domain gap between real and synthetic images is too large and the network cannot generalize to the real data. Since enhancing the realism of the procedural model is a very time-consuming task, we first try faster changes to shrink to domain gap. We add a second, slightly defocused branch to the background of each image without changing the branch generation itself. This is done to mimic the cluttered environment of the greenhouse and to make the network invariant to camera focus. In total, we render $\left|S_{2}\right|\;=\;2472$ new images. We also increase the augmentation in *h*_2_: Referring to **Figure 7**, we add Gaussian blur, contrast adjustment, hue shifting, and additive Gaussian noise, and retrain the network. This indeed improves the detected of infected leaves, but not by an sufficient amount as shown in **Figure 8**.

### 5.3 Iteration k=3

Since increasing the augmentation is cheap and gave good results in the previous iteration, we now increase it even further for *h*_3_. We continue to use the same operations but with a larger variety of parameters. As seen in **Figure 7**, the look of the images is quite drastically altered now. The dataset remains the same as in the previous iteration, thus $\left|S_{3}\right|\;=\;\left|S_{2}\right|$ . After retraining the network, we find that the accuracy improves only marginally (**Figure 8**), indicating that we have to proceed in a different direction.

### 5.4 Iteration k=4

It is now clear, that our synthetic images are too different from the real photos. However, many things could be improved about the renderings: We could have more variety in the branch geometry, increase texture details, or model more complex scenes (e.g. creating geometry for the whole greenhouse and a large amount of plants instead of a single branch in front of background panoramas). Implementing all of these improvements would be prohibitively costly, so instead, we perform a detailed analysis on which images are classified well. Since healthy plants are usually classified as healthy, we focus on images of infected plants.

**Figure 9:**
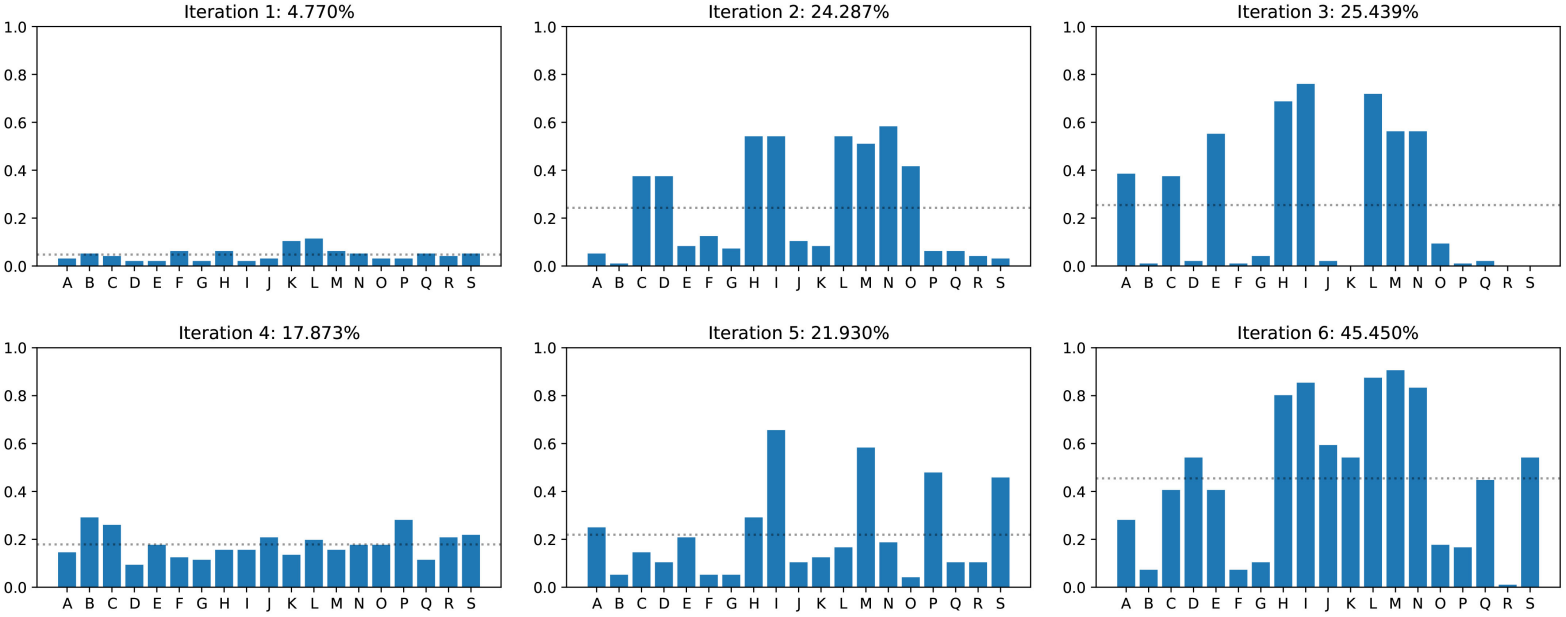
Network accuracy on real images of infected plants for each iteration. The letters on the horizontal axis denote the individual images. The dotted line shows the average accuracy across all images.

**Figure 10:**
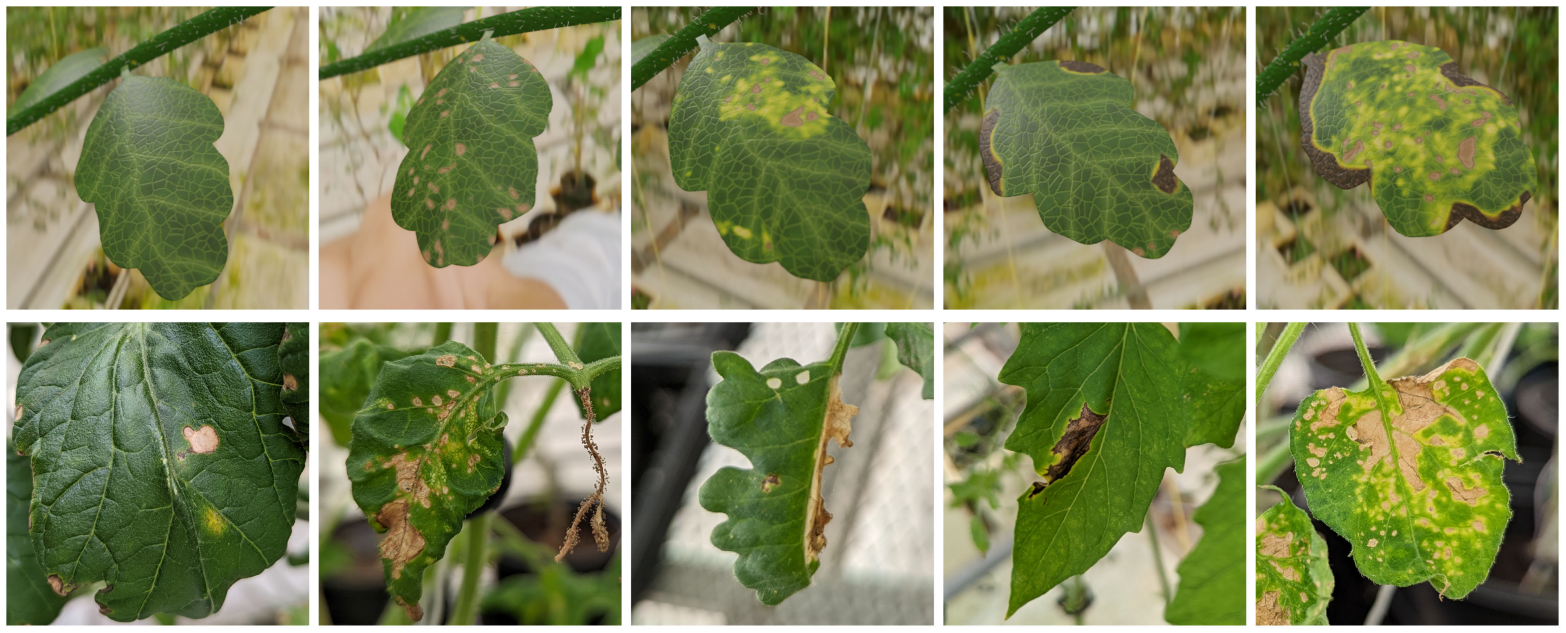
Different types of diseases affecting the leaf texture of these tomato plants (*Solanum lycopersicum*). Top row: Synthetic images. Bottom row: Photographs.

**Figure 9** shows the accuracy for 19 different input images across the iterations. We find that the distribution is extremely uneven: Some images are repeatedly classified correctly while others are almost never. Comparing the real input images with our renderings (see **Figure 10**), we find that infection can alter the leaf textures in many different ways. Infections resembling the type that we initially modeled are then classified correctly, while other types of patterns are not detected. We therefore improve our texture creation pipeline by adding additional disease types. The new synthetic disease textures are also shown in **Figure 10**. Since we have significantly increased the variety of the dataset, we increase to total number of images to $\left|S_{4}\right|\;=\;6400$ . The hyperparameters stay the same, thus $h_{4}=h_{3}$ .

While training the network we find that the accuracy even on synthetic data is very low. The increased diversity in the dataset makes the training significantly harder.

### 5.5 Iteration k=5

To make the training easier without reducing the diversity of the dataset, we reduce the image augmentation in *h*_5_ again to the previous level as in *h*_2_. The dataset stays the same as in the previous iteration, thus $\left|S_{5}\right|\;=\;\left|S_{4}\right|$ . The training works and we get an overall accuracy similar to iteration 3 (**Figure 8**). However, when looking at the performance of individual images (**Figure 9**), we find that the distribution is more even than for iteration 3. This means, that the modeling of additional diseases has paid off.

### 5.6 Iteration k=6

We attribute the remaining inaccuracies in the classification to the different global look of the renderings and photos. This could be addressed by stronger augmentation, however in iteration 4 we saw that a too difficult dataset makes the training harder. We therefore change the training strategy in *h*_6_ and employ a mixed training model, without changing the dataset $\left(\left|S_{6}\right|\;=\;\left|S_{5}\right|\right)$ . During the first half of the training, medium augmentation as in *h*_2_ is used. Once the network works sufficiently well, we switch over the stronger augmentation of *h*_3_. This results in an initial drop of the accuracy (since the problem became harder), but eventually the half trained network can adjust to the stronger augmentation and reach a high accuracy on them.

### 5.7 Improving the classification results

**Figure 11:**
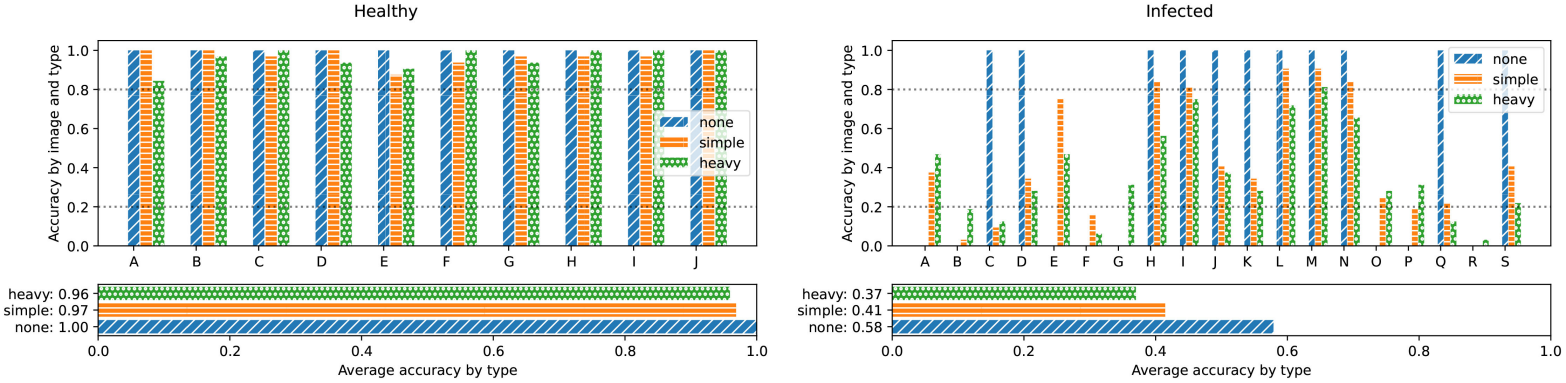
Network prediction performance by input image for different augmentation modes. For each mode, the input image was augmented 32 times with random parameters according to the given augmentation mode. The number of correct classifications are added up. The vertical scale shows normalized accuracy. Note, that since the *none* augmentation mode does not alter the image, either all or no instances of the image are correctly classified since the network is deterministic.

After the network training, we now perform a deeper statistical analysis of the results. During the training with synthetic renderings, we used augmentation to increase the diversity and to mimic artifacts found in real images but not the renderings (blurring, noise, etc.). As seen in **Figure 7**, the augmentation can be quite strong. Therefore, when using the trained network for classification, the same augmentation as during the training should be applied before passing the images to the network. However, since the photos already contain artifacts mimicked by the augmentation, this would in some sense result in a double augmentation. To decide, which augmentation mode should be applied for real images, we perform an analysis, where each image is classified multiple times (since the augmentation parameters are chosen at random, every time). The results are shown in **Figure 11**.

We find, that there is no clear best performing augmentation mode for real input images but that the results rather depend on the input image. We further find, that healthy input images are classified as healthy in over 80% of the cases, independent from the augmentation mode. Furthermore, for 16 out of the 19 infected images, at least one augmentation mode lies above the equivalent threshold (20%). We conclude, that the network is biased towards the healthy case. But by taking the estimated accuracy into account, this bias can be corrected. If we consider for any input image a value of below 80% healthy score (equivalent to an above 20% infected score) as infected, then 26 out of 29 images are correctly classified. We therefore reach an overall accuracy of 89.6%, which is roughly equal to the initial $q_{\min}$ , ending our optimization. Without this analysis, the naive threshold would be at 50%, leading to an accuracy of only 75%. In some sense, we apply a post-processing to the network’s output to increase the accuracy – similar to the pre-processing of the inputs in the augmentation step.

### 5.8 Further comments

A summary of the cost and performance of each iterations is shown in **Figure 8**. It is important to note, that the cost types are separated into two distinct categories. $C^{E}$ and $C^{M}$ directly relate to working hours of an expert and are thus typically very expensive. In contrast, $C^{R}$ and $C^{T}$ relate to computational time of computers. For scenarios like our use-case, they can often run over night and thus do not stall the general development if scheduled carefully. However, for larger datasets and more complicated training $C^{R}$ and $C^{T}$ can also become very expensive, for instance, the training cost of the recently released *Stable Diffusion* network [Rombach et al. (2022)] was about USD 600000.^^2^^

In this use-case we trained a network to the desired accuracy in only *n* = 6 iterations. Out of those, only one included a redesign of the dataset. We can see several key points here: Firstly, the dataset is not optimized for photorealism but rather for the distinction between healthy and infected leaves. Secondly, this crucial information became available through thorough evaluation. In other words, increasing $C_{k}^{\text{E}}$ can over proportionally decrease $C_{k}^{\text{M}}$ , $C_{k}^{\text{R}}$ , and $C_{k}^{\text{T}}$ , leading to an overall lower cost *C*. And thirdly, a good understanding of the behavior of the trained network can be used to increase its performance.

Using the presented development model, total costs of about *C* = 125.5 h have been invested to develop our classifier comprising approximately CE+CM= 64 h of human work and 61.5 h of computation. For comparison, we estimate the total costs of human work without applying the presented development model. Based on our extensive previous work comprising plant modeling, simulation, and rendering, we estimate the development time of a fully photo-realistic plant generator to be at least three months for a single expert. In this unguided approach, no continuous, quantitative feedback based on the intermediate network performance would be provided during the development and thus all visual features would be addressed with equal importance. This lack of prioritization then severely impacts the efficiency, driving up overall development cost.

### 5.9 Comparison to real training data

**Figure 12:**
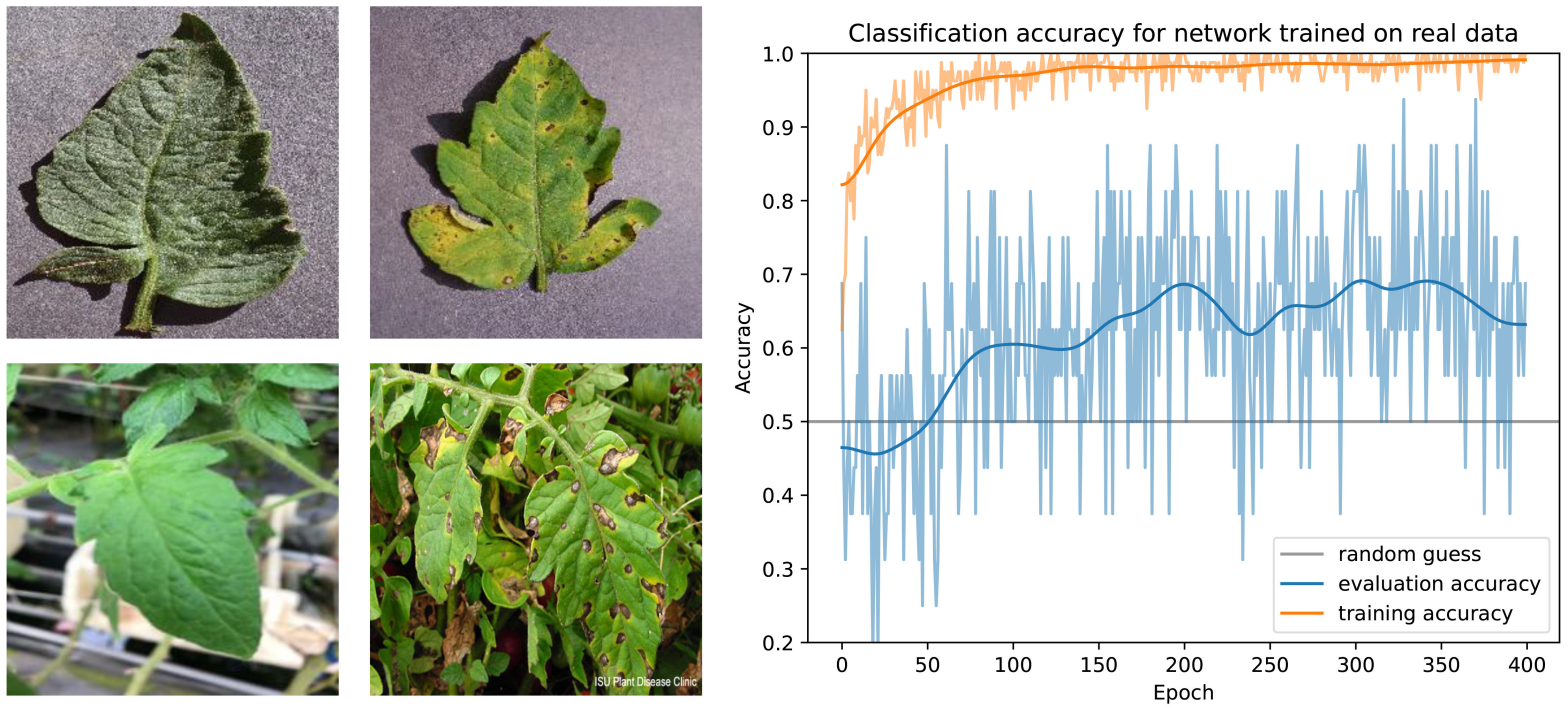
Training results for a real dataset. Left: Example images from the dataset. Right: Classification results during the training for images from the dataset (orange) and our own images from the greenhouse (blue).

The baseline alternative for the creation of a synthetic dataset is the use of the best available real dataset. Contrary to the specifically designed synthetic data, these real image are photorealistic by definition but may not fit the task domain as closely as a custom dataset. The popular *Plant Village* dataset ([Hughes and Salathe (2015)]) contains around 5500 pictures of healthy and infected tomato leaves, albeit detached from the plant and lying on a gray background. We therefore use a community extension of it that also contains leaves in their natural environment^3^. Example images of this dataset are shown in **Figure 12**.

We use this dataset to train the same network that was used for the synthetic training data. We find that classification results on the validation dataset are very accurate, indicating that the network was properly trained (see **Figure 12** for the training curves). However, even after extensive training, classification results on our greenhouse photos are barely better than random guessing, indicating poor generalization.

The most promising steps for improving the performance are directed towards overcoming the domain gap between training and evaluation images - which in turn means collecting and labeling a significant amount of additional photographs.

## 6 Conclusion

In this paper, we have presented a development model for the development of synthetic training data in order to efficiently automatize agricultural tasks with ML, using tomato leaf disease detection in a greenhouse as a case study. We demonstrated, that hand-designed synthetic training data outperforms real training data collected for a different task, justifying future development in this direction. While it is to some extend straightforward to create “good” training data, it is much more difficult to do so in a cost efficient way. We have demonstrated that by using our development model the desired goal can be achieved by a small amount of only six iterations in our use-case. Importantly, we find that photorealism (which is expensive to achieve) is not the main quality driver of the trained network. Rather, most iteration steps consist only of small changes that optimize the data for the distinction between the different classes, rather than overall realism. Naturally, our development model is driven by a human expert. It is therefore less of a plug-and-play solution but rather a development philosophy enabling the efficient and effective use of synthetic data. In future work, we aim to further automatize different steps within the development process to boost efficiency and reduce the time spent by the human in the loop.

Based on our development model, a neural classifier could be efficiently developed for the early detection of infections in our greenhouse complex growing tomato plants (*Solanum lycopersicum*). Total costs of about 125.5 h have been sufficient to develop the classifier within our development model which only includes approximately 64 h of human work (evaluation plus modeling costs) and 61.5 h of computation (rendering plus training costs). Note, that these costs are only a very small fraction of the effort of the research project presented here as – next to the formalization of the development model which emerged from the experience with different use cases – we developed the corresponding technical routines to allow for an efficient workflow. Also not included is the training time of the developer who has to become familiar with these routines and working within the presented development model. Our classifier performs with an accuracy of about 90% significantly reducing the need for manual checking of the entire greenhouse complex. Using UAVs, our final early disease detection method for tomato plants can be implemented in greenhouse complexes at low costs. However, our classifier comes with limitations as infected leaf textures have been generated from healthy ones by adding typical patterns of dead leaf cells. If, e.g., a disease is mainly visible at an early stage by looking at the branches instead of the leaves, it is not sufficient to only focus on leaf textures, but instead more investments have to be made to model the implications on the branches. This could require the modeling of wilting effects influencing the whole plant geometry and not only the leaves’ textures. This is why, among others, we aim for an efficient simulator of plant wilting in future work addressing geometrical features of plant diseases in addition to those which could already be modeled by modifying leaf textures.

## Figures and Tables

**Figure A1:**
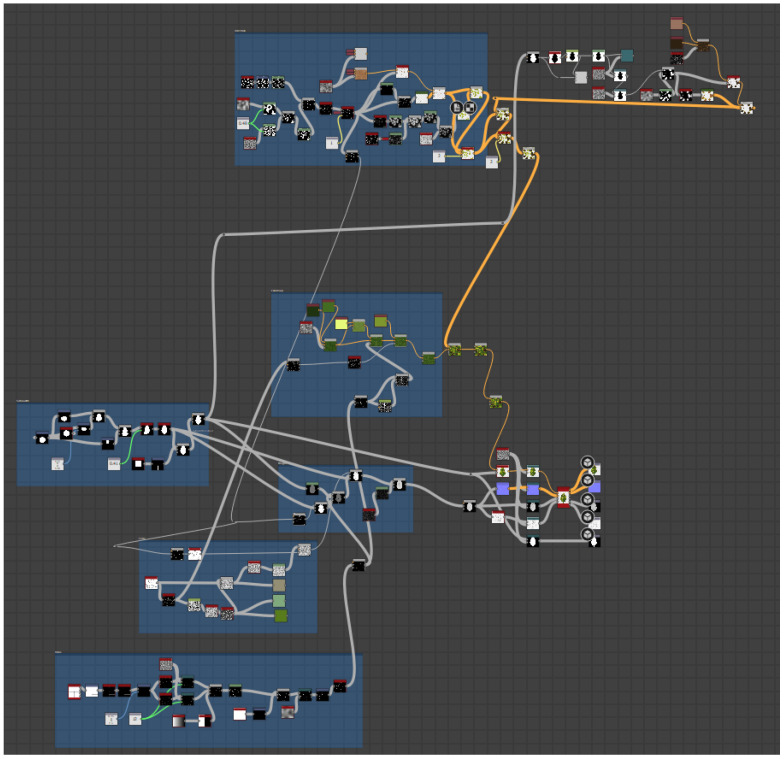
A screenshot from *Adobe Substance 3D Designer*, showing a highlevel overview of the structure and complexity of our node setup for texture generation.

**Figure A2:**
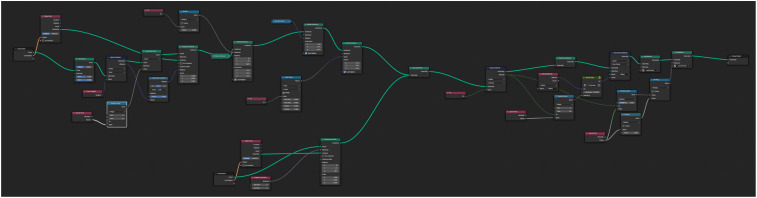
A screenshot from *Blender*, showing a highlevel overview of the structure and complexity of our node setup for tomato branch generation.

## Data Availability

The raw data supporting the conclusions of this article will be made available by the authors, without undue reservation.

## Footnotes

1 For comparison, the 2013 movie *Gravity* would have required 7 000 years of rendering time on a single personal computer available at that time; see https://creativechair.org/chris-parks/.

2 See https://x.com/emostaque/status/1563870674111832066.

3
https://www.kaggle.com/datasets/cookiefinder/tomato-disease-multiple-sources.

## 1 Appendix

### 1.1 Texture generation

All textures are generated using *Adobe Substance 3D Designer*, a node-based procedural modeling tool. Our node graph, shown in **Figure A1** consists of a total of 155 nodes that first model the biological structure (bumps, veins, infected parts, etc.) and then derive the various textures (basecolor map, normal map, occlusion map height map, and alpha map) from it.

Many node types rely on input noise. To generate different textures, we use a Python script that randomly changes the initial seed of the noise generation as well as randomly enabling/disabling various disease types.

The file is available on our project website or upon contacting the authors.

### 1.2 Geometry generation and rendering

Our 3D scenes are modeled inside Blender, using the generated textures from the previous step. Leaves are modeled as rectangular spline surfaces that is overlayed with the alpha texture to create the accurate leaf outline. A spline with randomly altered control points models the branch to which the leaves are then attached. Depending on the generated class, either *healthy* or *infected* textures are used for the leaf materials. In some scenes, additional branches with random classes are put into the background to increase scene depth. Using appropriate depth of field parameters, these appear blurred and the network learns to ignore them. For the background images, we use random panorama photographs collected inside a greenhouse. The node setup inside *Blender* is shown in **Figure A2**.

Scene generation takes around 1 second, while rendering takes around 3 seconds. Although both numbers could likely be optimized, we found the overall generation time sufficiently fast and did not feel that time spend optimizing this would pay off in the long run.

The project file is available on our project website or upon contacting the authors.

### 1.3 Network architecture

Our classifier is implemented in *Python* using the *tensorflow* and *keras* frameworks as well as *imgaug* for augmentation. The network architecture is defined in the framework through the following commands:

model = tf.keras.models.Sequential()
model.add(tf.keras.layers.Conv2D(64, (3, 3), input_shape=(256, 256, 3)))
model.add(tf.keras.layers.BatchNormalization())
model.add(tf.keras.layers.Activation(‘relu’))
model.add(tf.keras.layers.MaxPooling2D(pool_size=(2, 2)))
model.add(tf.keras.layers.Conv2D(64, (3, 3)))
model.add(tf.keras.layers.Activation(‘relu’))
model.add(tf.keras.layers.BatchNormalization())
model.add(tf.keras.layers.MaxPooling2D(pool_size=(2, 2)))
model.add(tf.keras.layers.Conv2D(128, (3, 3)))
model.add(tf.keras.layers.Activation(‘relu’))
model.add(tf.keras.layers.BatchNormalization())
model.add(tf.keras.layers.MaxPooling2D(pool_size=(2, 2)))
model.add(tf.keras.layers.Conv2D(128, (3, 3)))
model.add(tf.keras.layers.Activation(‘relu’))
model.add(tf.keras.layers.BatchNormalization())
model.add(tf.keras.layers.MaxPooling2D(pool_size=(2, 2)))
model.add(tf.keras.layers.Conv2D(256, (3, 3)))
model.add(tf.keras.layers.Activation(‘relu’))
model.add(tf.keras.layers.BatchNormalization())
model.add(tf.keras.layers.MaxPooling2D(pool_size=(2, 2)))
model.add(tf.keras.layers.Flatten())
model.add(tf.keras.layers.Dense(256))
model.add(tf.keras.layers.Activation(‘relu’))
model.add(tf.keras.layers.BatchNormalization())
model.add(tf.keras.layers.Dense(128))
model.add(tf.keras.layers.Activation(‘relu’))
model.add(tf.keras.layers.BatchNormalization())
model.add(tf.keras.layers.Dense(64, name=“feature_layer”))
model.add(tf.keras.layers.Activation(‘relu’))
model.add(tf.keras.layers.BatchNormalization())
model.add(tf.keras.layers.Dense(2))
model.add(tf.keras.layers.Activation(‘softmax’))

A graphical representation of this is shown in **Figure 5**.
