# Supplementary figures and images for: Synthetic data at scale: a development model to efficiently leverage machine learning in agriculture
Source: Front Plant Sci. 2024 Sep 16;15:1360113. doi: 10.3389/fpls.2024.1360113 (PMC11439777; doi:10.3389/fpls.2024.1360113)

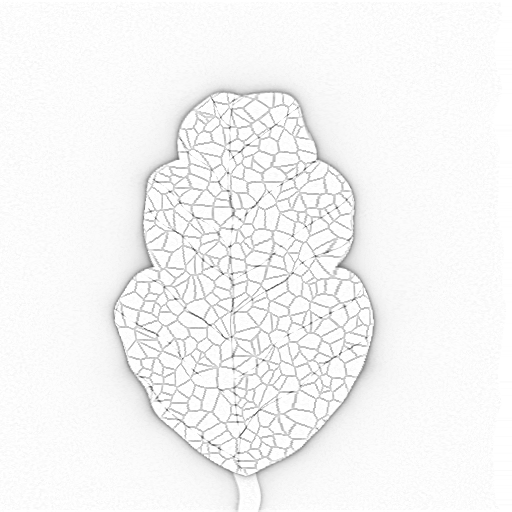

Supplement: Supplementary file 1 [file DataSheet1.zip › Leaf Textures/test_dataset/healthy/000_Tomato_Leaf_ambientOcclusion.png]

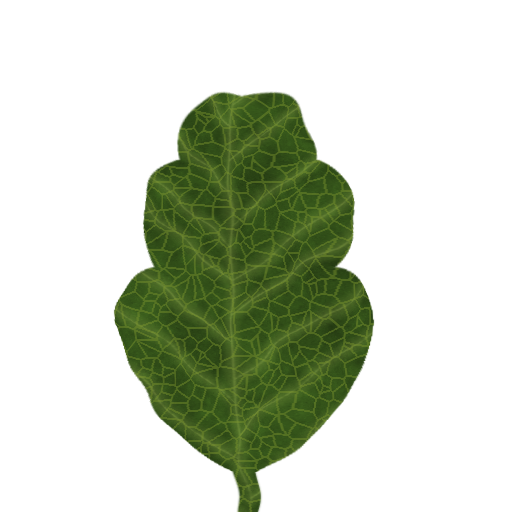

Supplement: Supplementary file 1 [file DataSheet1.zip › Leaf Textures/test_dataset/healthy/000_Tomato_Leaf_basecolor.png]

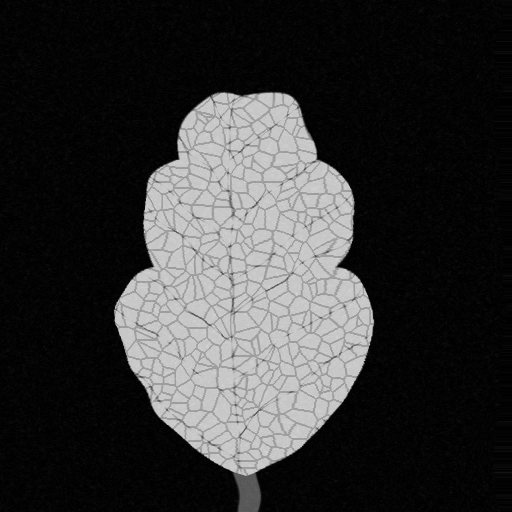

Supplement: Supplementary file 1 [file DataSheet1.zip › Leaf Textures/test_dataset/healthy/000_Tomato_Leaf_height.png]

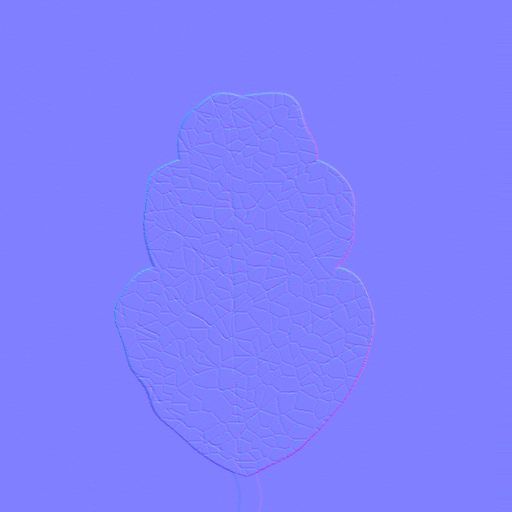

Supplement: Supplementary file 1 [file DataSheet1.zip › Leaf Textures/test_dataset/healthy/000_Tomato_Leaf_normal.png]

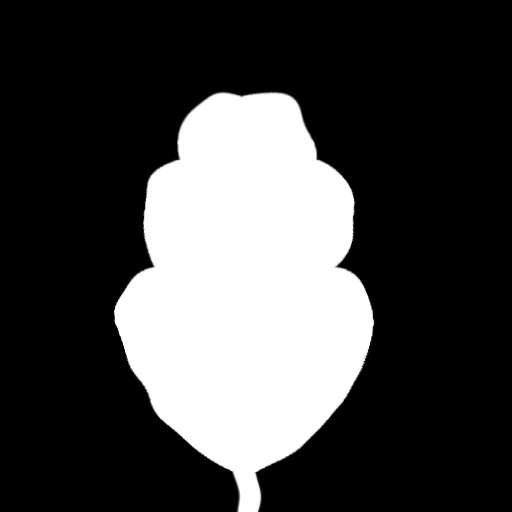

Supplement: Supplementary file 1 [file DataSheet1.zip › Leaf Textures/test_dataset/healthy/000_Tomato_Leaf_output.png]

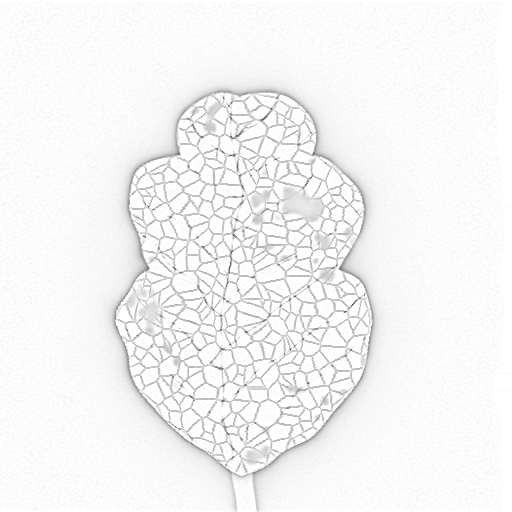

Supplement: Supplementary file 1 [file DataSheet1.zip › Leaf Textures/test_dataset/infected/004_Tomato_Leaf_ambientOcclusion.png]

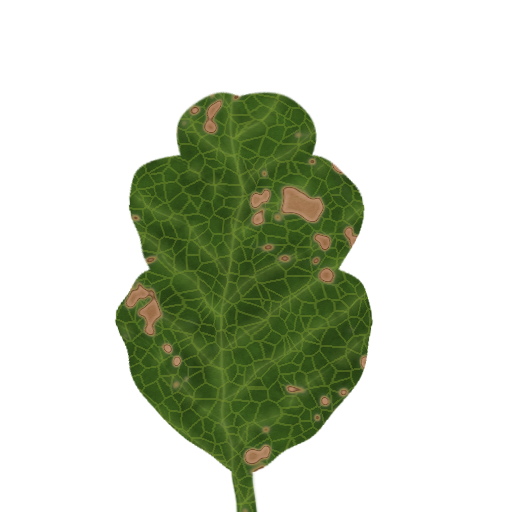

Supplement: Supplementary file 1 [file DataSheet1.zip › Leaf Textures/test_dataset/infected/004_Tomato_Leaf_basecolor.png]

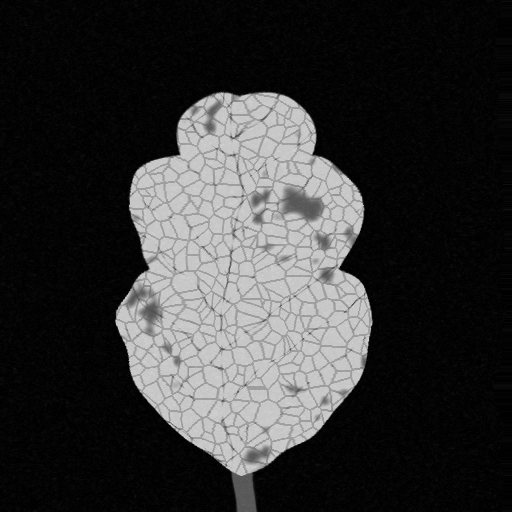

Supplement: Supplementary file 1 [file DataSheet1.zip › Leaf Textures/test_dataset/infected/004_Tomato_Leaf_height.png]

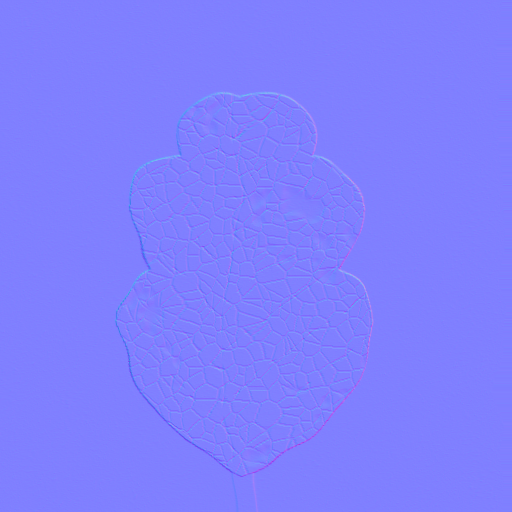

Supplement: Supplementary file 1 [file DataSheet1.zip › Leaf Textures/test_dataset/infected/004_Tomato_Leaf_normal.png]

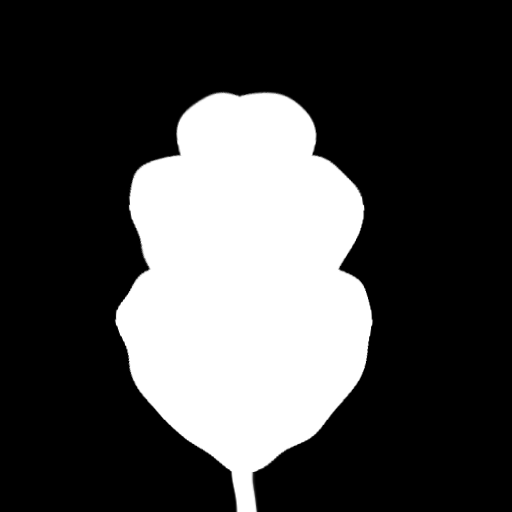

Supplement: Supplementary file 1 [file DataSheet1.zip › Leaf Textures/test_dataset/infected/004_Tomato_Leaf_output.png]

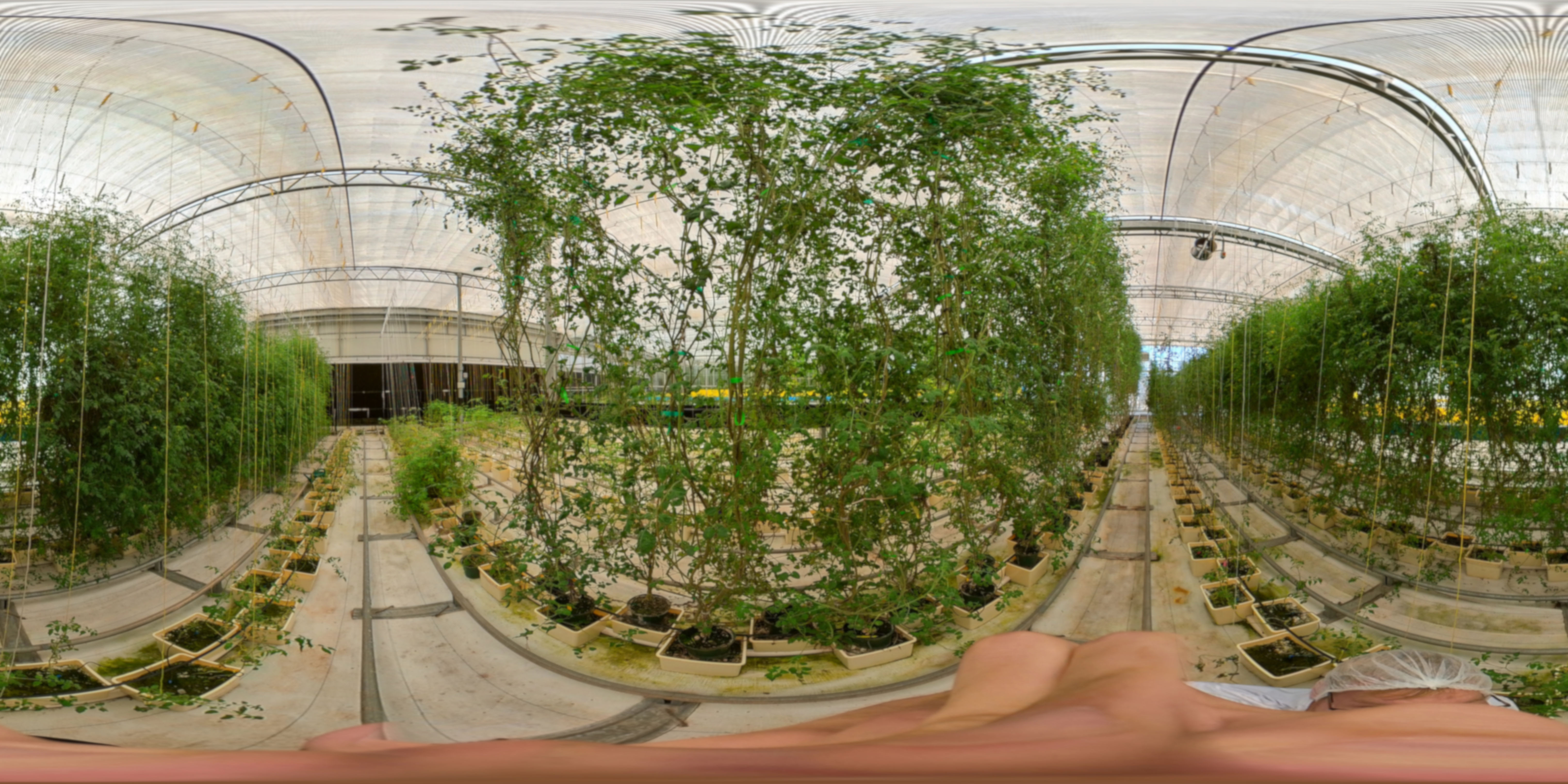

Supplement: Supplementary file 1 [file DataSheet1.zip › Plant Images/env/01.jpg]

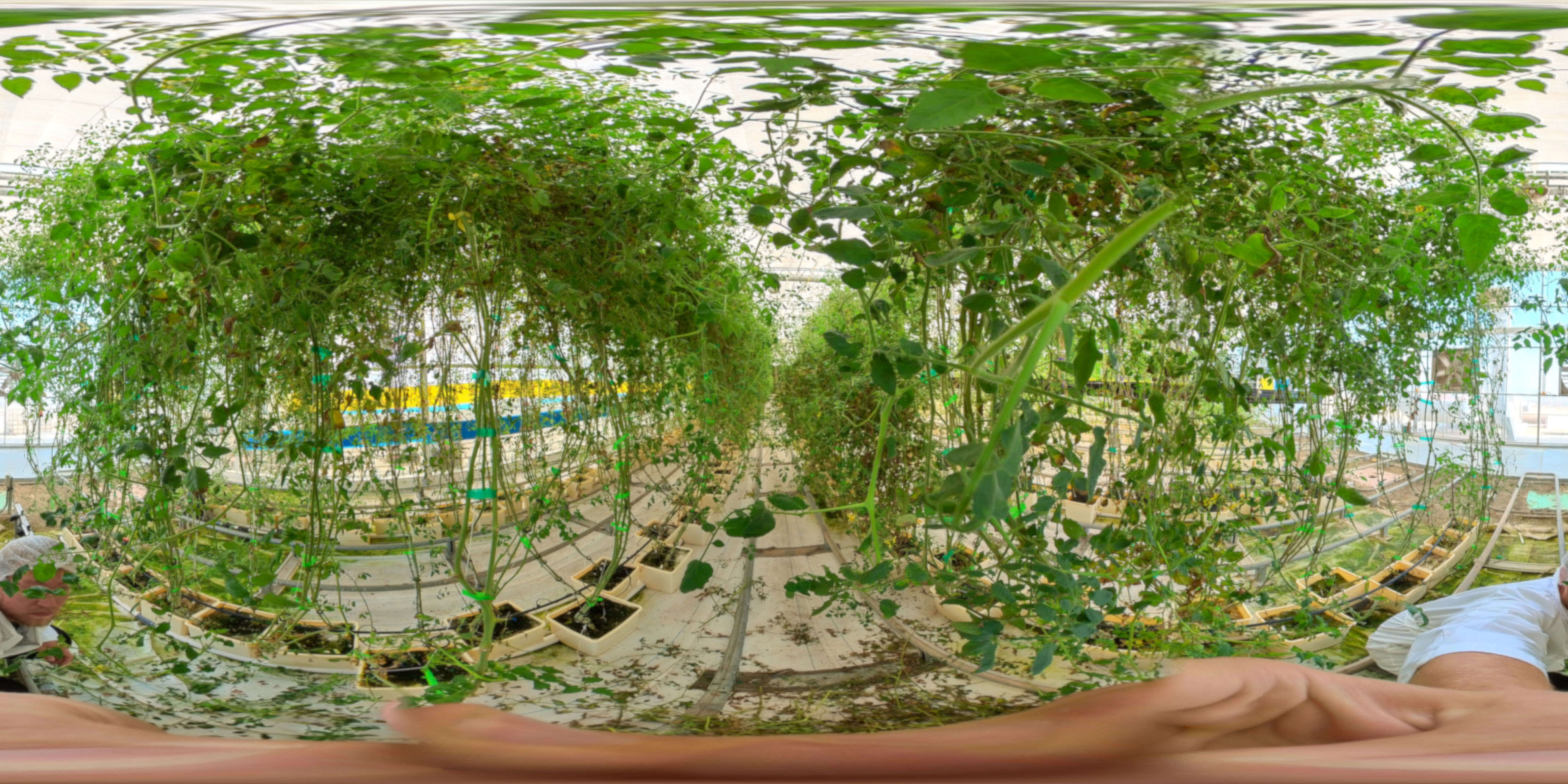

Supplement: Supplementary file 1 [file DataSheet1.zip › Plant Images/env/02.jpg]

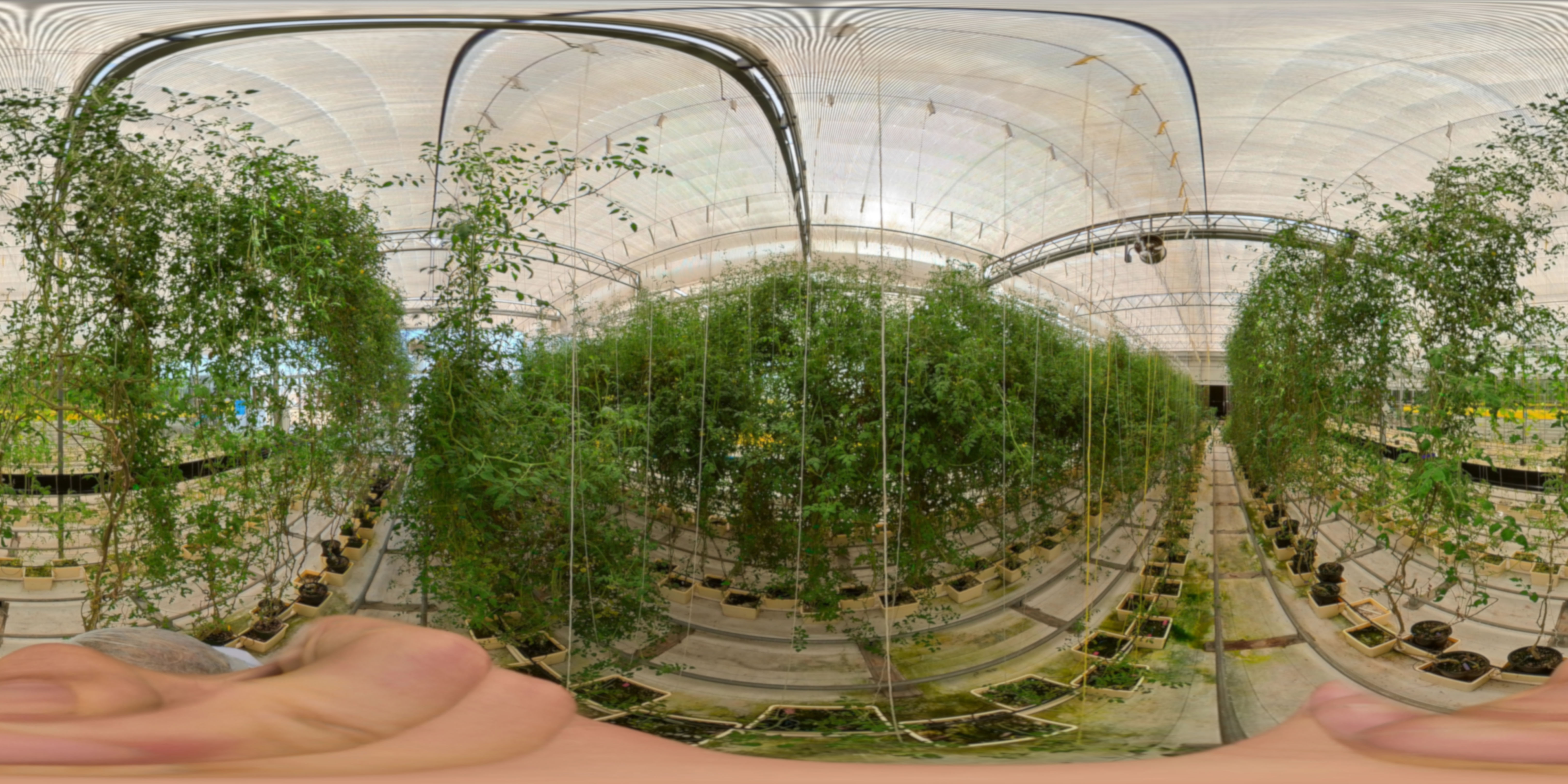

Supplement: Supplementary file 1 [file DataSheet1.zip › Plant Images/env/03.jpg]

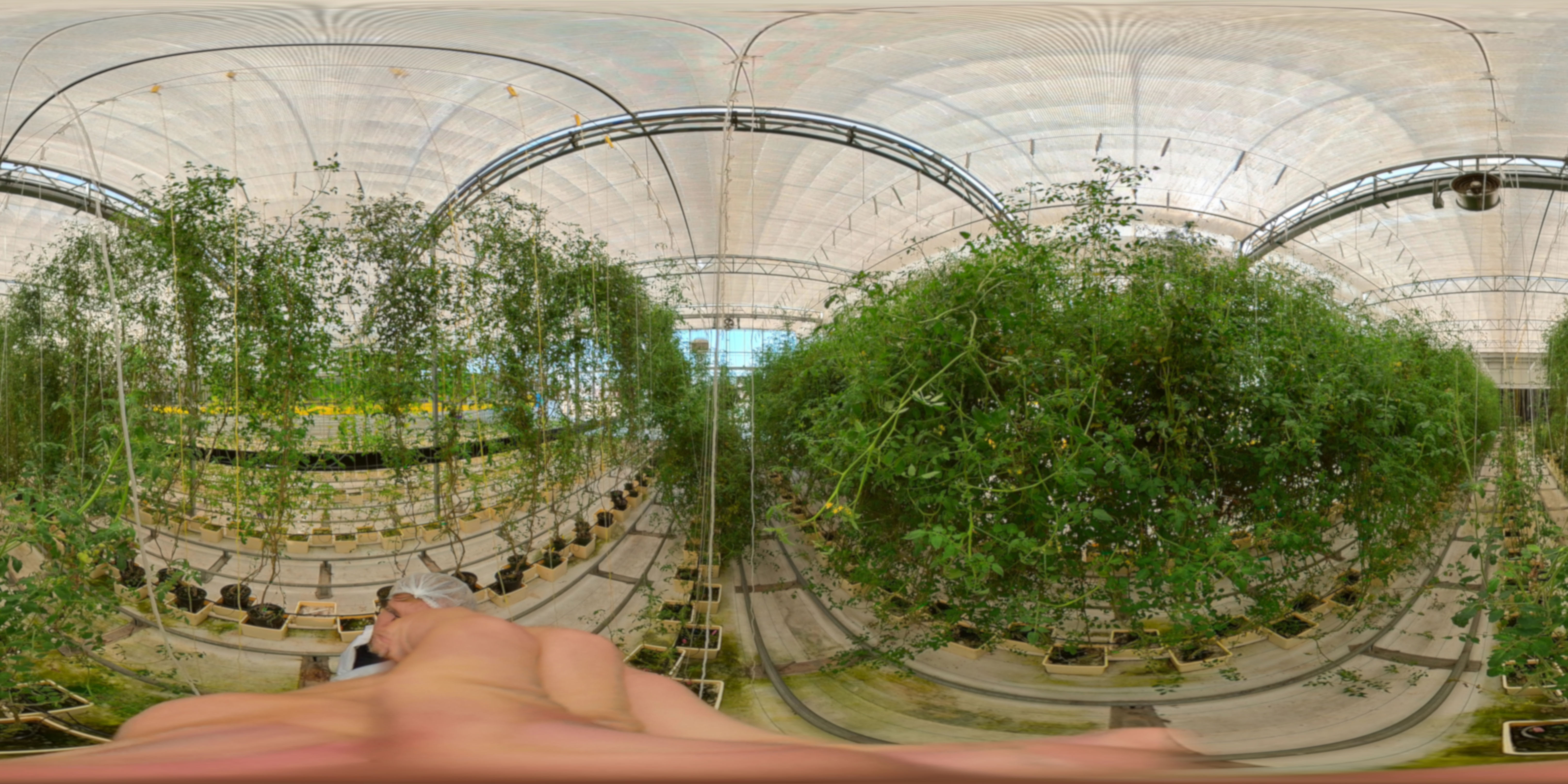

Supplement: Supplementary file 1 [file DataSheet1.zip › Plant Images/env/04.jpg]

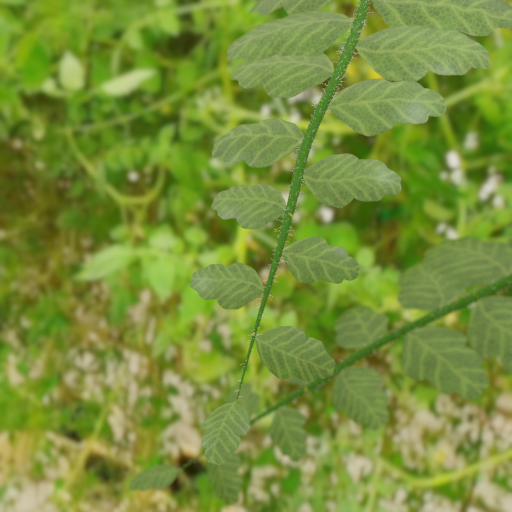

Supplement: Supplementary file 1 [file DataSheet1.zip › Plant Images/renderings/img0000.png]

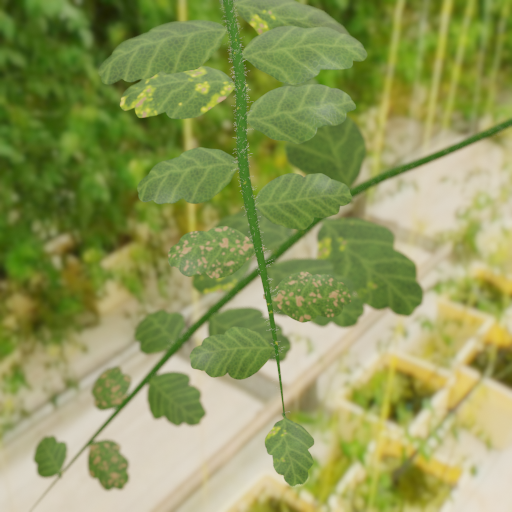

Supplement: Supplementary file 1 [file DataSheet1.zip › Plant Images/renderings/img0001.png]

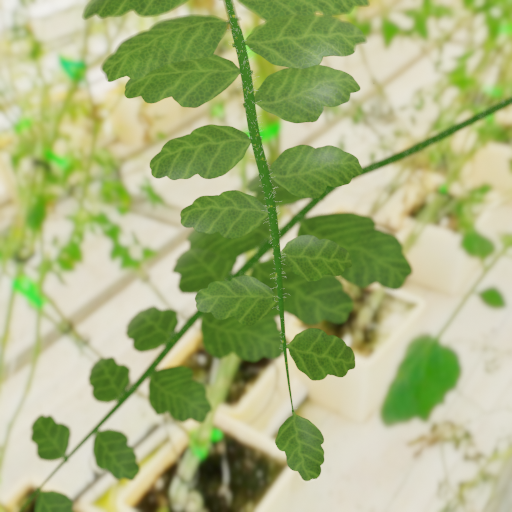

Supplement: Supplementary file 1 [file DataSheet1.zip › Plant Images/renderings/img0002.png]

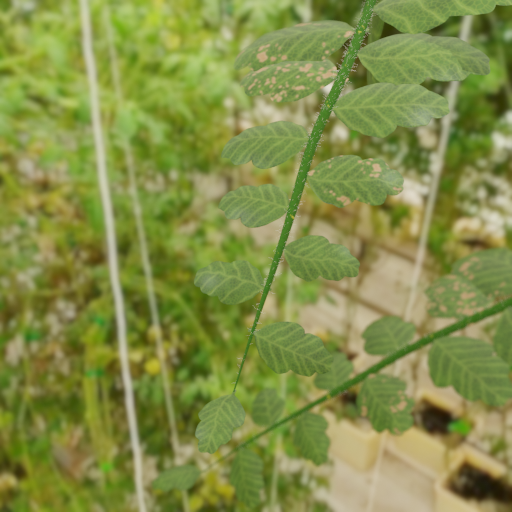

Supplement: Supplementary file 1 [file DataSheet1.zip › Plant Images/renderings/img0003.png]

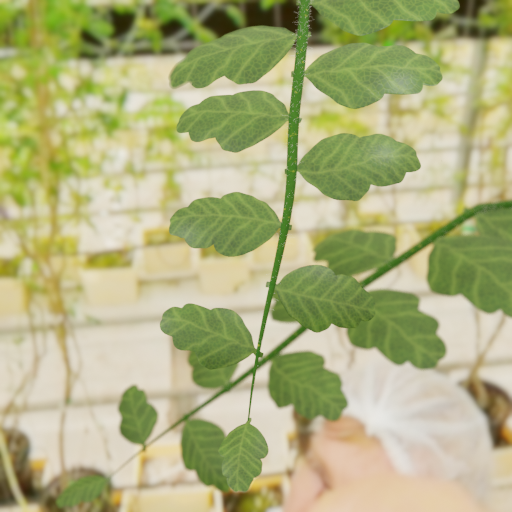

Supplement: Supplementary file 1 [file DataSheet1.zip › Plant Images/renderings/img0004.png]

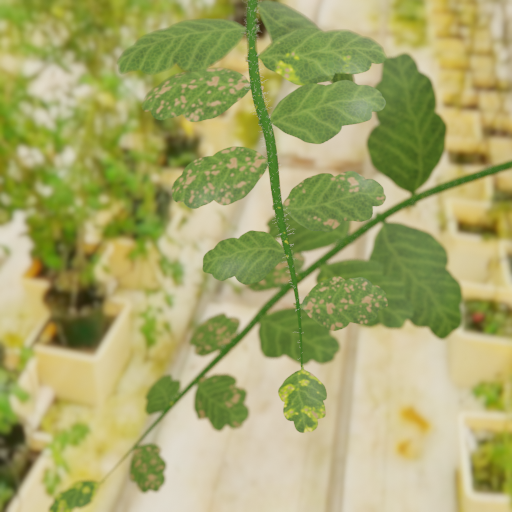

Supplement: Supplementary file 1 [file DataSheet1.zip › Plant Images/renderings/img0005.png]

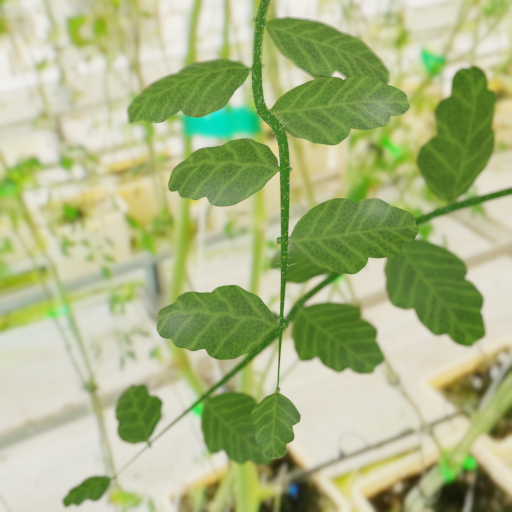

Supplement: Supplementary file 1 [file DataSheet1.zip › Plant Images/renderings/img0006.png]

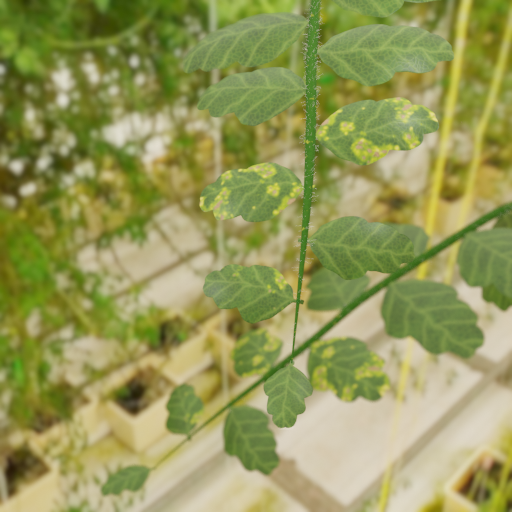

Supplement: Supplementary file 1 [file DataSheet1.zip › Plant Images/renderings/img0007.png]

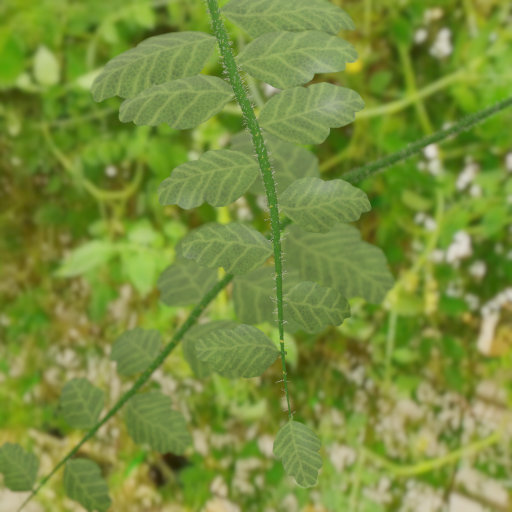

Supplement: Supplementary file 1 [file DataSheet1.zip › Plant Images/renderings/img0008.png]
